# Diversification of single-cell growth dynamics under starvation influences subsequent reproduction in a clonal bacterial population

**DOI:** 10.1093/ismejo/wrae257

**Published:** 2024-12-23

**Authors:** Sotaro Takano, Miki Umetani, Hidenori Nakaoka, Ryo Miyazaki

**Affiliations:** Bioproduction Research Institute, National Institute of Advanced Industrial Science and Technology (AIST), Tsukuba, 305-8566, Japan; Integrated Bioresource Information Division, Bioresource Research, Center, RIKEN, Tsukuba, 305-0074, Japan; Department of Basic Science, Graduate School of Arts and Sciences, The University of Tokyo, Tokyo, 153-8902, Japan; Research Center for Complex Systems Biology, The University of Tokyo, Tokyo, 153-8902, Japan; Universal Biology Institute, The University of Tokyo, Tokyo, 113-0033, Japan; Department of Optical Imaging, Advanced Research Promotion Center, Tokushima University, Tokushima, 770-8503, Japan; Bioproduction Research Institute, National Institute of Advanced Industrial Science and Technology (AIST), Tsukuba, 305-8566, Japan; Faculty of Life and Environmental Sciences, University of Tsukuba, Tsukuba, 305-0006, Japan; Computational Bio Big Data Open Innovation Laboratory (CBBD-OIL), AIST, Tokyo, 169-8555, Japan

**Keywords:** growth dynamics, single cell, starvation, time-lapse imaging

## Abstract

Most of the microbes in nature infrequently receive nutrients and are thus in slow- or non-growing states. How quickly they can resume their growth upon an influx of new resources is crucial to occupy environmental niches. Isogenic microbial populations are known to harbor only a fraction of cells with rapid growth resumption, yet little is known about the physiological characteristics of those cells and their emergence in the population. Here, we tracked growth of individual *Escherichia coli* cells in populations under fluctuating nutrient conditions. We found that shifting from high- to low-nutrient conditions caused stalling of cell growth with few cells continuing to divide extremely slowly, a process which was dependent on lipid turnover. Resuming high-nutrient inflow after low-nutrient conditions resulted in cells resuming growth and division, but with different lag times and leading to varying progeny. The history of cell growth during low-nutrient but not high-nutrient conditions was determinant for resumption of growth, which cellular genealogy analysis suggested to originate from inherited physiological differences. Our results demonstrate that cellular growth dynamics become diverse by nutrient limitations, under which a fraction of cells experienced a particular growth history can reproduce progeny with new resources in the future.

## Introduction

Control of reproduction rate is an important biological process for the adaptation to environmental change. Allocating a large amount of nutrient resources for reproduction can benefit organisms to grow their populations and occupy a niche, while it is not advantageous for survival under unfavorable growth conditions [1, 2]. This trade-off enables organisms to switch their physiologies depending on environmental conditions [3, 4]. Indeed, microorganisms often encounter environmental changes between favorable and unfavorable growth conditions (e.g. transitions from nutrient-rich to nutrient-poor conditions) and drastically change their growth rates. It is well known that bacterial cells actively proliferating in the host environment (e.g. human gut) rapidly lose their reproductivity when they are released from the body [5]. In contrast, microbes residing in nutrient-poor environments, such as soil, freshwater, and deep-sea sediment, which comprise the majority of the microbial community in nature, are occasionally exposed to short-term nutrient availability [6]. For those cells, not only how long they can survive in such poor conditions but also how quickly they can resuscitate themselves from the growth-arrested state by the supply of nutrients is key to increasing their fitness in fluctuating environments.

Bacterial cells exposed to starvation do not immediately restart their growth when nutrients are provided but only after a certain period—known as lag time. Individual cells vary in lag time even in isogenic bacterial populations [7], and population regrowth after starvation is primarily determined by a subset of cells with comparatively short lag times [8], suggesting specific phenotypic differences that determine rapid or slow resumption of growth. Despite its ecological significance, how such cellular heterogeneity in growth resumption emerges in a clonal bacterial population is poorly understood. In principle, both stochastic and non-stochastic processes can generate phenotypic heterogeneity in a cellular population. Stochastic physiological changes due to molecular noise (e.g. fluctuations in gene expression) can lead to increased survival [9–11], while there are some cases where cellular physiology in ancestral cells (i.e. non-stochastic) affects the fate of their descendant cells after several generations [12–15]. A previous study has reported that the population growth rate prior to starvation affects the reproductive success in subsequent nutrient upshift [16], implying that cellular growth history would be an important factor for determining the regrowth capability of bacterial cells. However, as our knowledge on bacterial growth dynamics during starvation is scarce at the single-cell resolution [17, 18], to what extent growth-related traits vary in a clonal population under fluctuating conditions has not been well characterized.

In this study, we use a custom microfabricated device to track the growth of *Escherichia coli* at single-cell resolution using time-lapse microscopy. We directly follow the emergence of heterogeneity in cellular growth dynamics upon a shift from high-nutrient to low-nutrient conditions and the subsequent variation in cellular reproduction with an additional carbon source. We then explore the interrelation between cellular growth history and reproduction by retrospectively tracing cell lineages and quantifying various cellular and spatial parameters. Characteristic growth history linked to the cellular reproduction is further examined by changing the length of the low-nutrient period. Finally, using a mutant lacking a gene for β-oxidation, a lipid-turnover process considered to provide starved cells with endogenous carbon and energy [19], we evaluate the effect of fatty acid metabolism on growth in the low-nutrient period and subsequent growth resumption. Our genealogical analysis reveals that cells showing higher reproduction after days of low-nutrient condition tend to be phylogenetically proximal, indicating that differences in cellular physiology emerged and inherited during the low-nutrient condition have an impact on future regrowth.

## Materials and methods

### Bacterial strains and media

We used *E. coli* strain MG1655 and its derivatives for all experiments in this study. To construct a fluorescent reporter strain for the detection of cell regions in image analysis, we amplified a 500 bp upstream region of *intS*, a constitutive *tetA* promoter (*P_tetA_*), *sfgfp*, *cat* (chloramphenicol acetyltransferase), and a 500 bp downstream region of *intS* by PCR using primers and templates described in Table S1. Those fragments were cloned in that order into pEMG [20] by NEBuilder HiFi DNA Assembly Master Mix (New England Biolabs), and used as a template to amplify a *P_tetA_*-*sfgfp*-*cat* fragment flanked by 50 bp of upstream and downstream regions of *intS*. We then introduced the fragment into the *intS* locus on the MG1655 chromosome by RED/ET recombinations (Gene Bridges GmbH, Germany). This strain (MG1655*ΔintS*::*P_tetA_*-*sfgfp*-*cat*) was designated as WT in this study. We also constructed the *ΔfadE* strain by inserting the Km cassette of JW5020-KC (NBRP Keio Collection, Japan) into the *fadE* locus on the WT chromosome using P1 transduction [21]. Bacterial strains were grown at 30°C in M63 minimal medium (62 mM K_2_HPO_4_, 39 mM KH_2_PO_4_, 15 mM (NH_4_)_2_SO_4_, 2 μM FeSO_4_ · 7H_2_O, 200 μM MgSO_4_ · 7H_2_O), where 0.02% of glucose was supplied when necessary.

### Fabrication of the well chamber

Microfabrication was conducted as described previously [22]. Briefly, we first made thin chromium (Cr) film on a coverslip via thermal evaporation by SVC-700TM (Sanyu, Japan). The Cr-coated coverslips were spin-coated by a positive photoresist AZP1350 (AZ Electronic Materials, Luxembourg). After soft baking for 1.5 min at 95°C, the photoresist-coated coverslips were irradiated by UV for 85 mJ/cm^2^ in total using a mask-aligner MA-20 (Mikasa, Japan) with a custom-made photomask. The UV exposed regions of photoresists were developed in NMD-3 (Tokyo Ohka Kogyo, Japan), and the uncovered region of chromium on the coverslip was etched by MPM-E350 (DNP Fine Chemicals, Japan). The coverslips were soaked in buffered hydrofluoric acid solution 110-BHF (Morita Kagaku Kogyo, Japan) for ~15 min at 23°C to etch uncovered part and then soaked in milliQ water to stop the reaction. The photoresist and Cr-layer remaining on the coverslips were removed by acetone and MPM-E350.

Chemical decoration of the coverslip and the cellulose membrane was performed as previously described [22]. We pre-coated microfabricated coverslips by 3-(2-aminoethylaminopropyl) trimethoxysilane (Shinetsu Kagaku Kogyo, Japan). EZ-Link NHS-LC–LC-Biotin (Thermo Fisher Scientific, MA, USA) was dissolved in phosphate buffer (0.1 mM, pH 8.0) and placed on the coverslip, and incubated at room temperature for four hours to immobilize biotin and the amino-group on the coverslip. A cellulose membrane Spectra/Por 7, MWCO 25000 (Repligen, MA, USA) was decorated by Pierce Streptavidin Hydrazide (Thermo Fisher Scientific, MA, USA). First, the membrane cut out to 3 cm square was soaked in milliQ water for 15 min and washed. This procedure was performed twice. Next, we treated the membrane with 0.1 M sodium periodate solution (FUJIFILM Wako, Japan) for 4 hours with gentle shaking and washed by milliQ water. Then, the membrane was soaked in 2 ml of streptavidin hydrazide solution in 0.1 mM phosphate buffer (pH 7.0) and gently shaken 14 hours at 25°C.

### Time-lapse microscopy

*E. coli* cells were precultured at 30°C for over 16 hours in M63 with 0.02% glucose and 40 μg/ml chloramphenicol. The preculture was diluted to OD_600_ = 0.02 with the fresh medium and incubated until OD_600_ = 0.1. A two μl of the culture was spotted onto the microchamber region on the coverslip, and covered by the streptavidin-decorated cellulose membrane. Then, a M63 agarose patch (~1 cm in diameter, solidified with 1.5% agarose) was placed on the top of it to facilitate bonding between biotin (decorated on the coverslip) and streptavidin (decorated on the cellulose membrane). After incubation at 30°C for 1 hour, the agarose patch was removed and a 20 μl of M63 with 0.02% glucose was spotted on the cellulose membrane to prevent drying. A polydimethylsiloxane pad with bubble trap groove for medium perfusion was fabricated as previously [23] and attached to the microchamber region on the coverslip via a double-sided frame-seal (BioRad, USA). This device was placed onto the stage of the microscope and connected to a syringe (Terumo, Japan) by 1 × 2 mm silicone tubes (AS ONE, Japan). Using a CX07100 syringe pump (ISIS, Japan), we first flowed M63 with 0.02% glucose at the rate of 15 ml/h for 20 min to fill a device and tubes with the medium, and then changed the rate to 1 ml/h. When we changed the culture condition from the presence to the absence of glucose, we replaced the medium with M63 without glucose at the flow rate of 15 ml/h for 20 min to instantaneously wash out the previous medium in the device, changed the rate to 1 ml/h for 6 hours, and then stopped perfusion. After 3 days, we switched the medium to M63 with 0.02% glucose at the flow rate of 15 ml/h for 20 min and then at the rate of 1 ml/h for the rest of the time.

Time-lapse images were obtained using a Zeiss AxioObserver inverted microscope equipped with Axiocam 506 mono CCD, 100×/1.40 oil Plan-Apochromat lens, and Colibri LED excitation light source (Carl Zeiss, Germany). A motorized stage unit and Definite Focus (Carl Zeiss, Germany) were used for capturing images at different positions on the device and stabilizing Z-offset in each position during the time-lapse measurements. The microscope and device assembly were enclosed in an acrylic box with a heater unit (Tokken, Japan) to keep the environment at 30°C constantly during the experiment. The motorized stage, fluorescence excitation, and image acquisition was controlled with ZEN software (Carl Zeiss, Germany). Cells were exposed to transillumination LED light for 200 ms for phase contrast and 470 nm LED light for 60 ms at 15% power for fluorescence images. The time-lapse interval was 10 min for the initial 24 hours and the last 10 hours of the experiments and 20 min for the rest of the time. We started to capture time-lapse images from 60 min after the start of the perfusion, where the culture condition in the microchamber became stable.

### Image processing and cell tracking

We used Schnitzcells [24], a MATLAB-based software (MathWorks, USA) for detecting cell contours, tracking cell lineages, and estimating cell size. We used sfGFP fluorescence images for the detection of the cellular region and making mask images. Using these mask images, we tracked individual cells across multiple images by the “trackcomplete” function in Schnitzcells, and obtained information of individual cells (e.g. birth time, division time, cell area) and genealogical information of cell lineages. These data were further processed by custom-made MATLAB scripts.

### Estimation of size increase rate

Using the data of cell area (i.e. number of pixels recognized as a cellular region converted to μm^2^ unit) of individual cells across all frames and their genealogical information, we calculated the size increase rate at the cell (denoted as *r_cell_*), subtree (denoted as *r_subtree_*), or lineage (denoted as *r_lineage_*) levels. In *E. coli* proliferation, both the number of cells and the size of individual cells have been reported to increase exponentially [25], and thus we checked whether the exponential growth is also applicable for our single-cell experimental data calculated in a cell area unit (μm^2^). In the high-nutrient period, a size increment of a cell in a given time frame ($\Delta{s}_i={s}_i\left(t+\Delta t\right)-{s}_i(t)$) linearly increased by initial cell size (${s}_i(t)$) (Fig. S1), indicating that *E. coli* cells grew exponentially rather than linearly as ${s}_i(t)={s}_0{e}^{r_{cell}t}$ (where *r_cell_* is the size increase rate constant and ${s}_0$ is the initial cell size). Therefore, the size increase rate of a cell ${r}_{cell}(t)$ at time *t* in a given time frame $\Delta t$ could be approximated as


(1) \begin{equation*} {r}_{cell}(t)=\frac{1}{\Delta t}\bullet \log \left(\frac{s\left(t+\Delta t\right)}{s(t)}\right) \end{equation*}

We further calculated the moving mean of ${r}_{cell}(t)$ within *∆f* (frames) of timepoint *t* as follows.


(2) \begin{equation*} \overline{r_{cell}(t)} = \frac{1}{\Delta f}\#\sum_i{r}_{cell}\left(t+i\Delta{t}^{\prime}\right)\#, for-\frac{\Delta f-1}{2}\le i\le \frac{\Delta f-1}{2} \end{equation*}

Here, $\Delta{t}^{\prime }$ is the time interval between frames (The definition of each parameter is described in Fig. S2A). Then, a ${r}_{cell}$, average of $\overline{r_{cell}(t)}$ from its birth or start of the period (*t* = *t_start_*) to division or end of the period (*t = t_end_*) as follows.


(3) \begin{equation*} {r}_{cell}\ =\frac{1}{f_{t_{end}}-{f}_{t_{start}}}\#{\sum}_{t={t}_{start}}^{t={t}_{end}}\overline{r_{cell}(t)} \end{equation*}

For the cells that were born before the period of interest, *t_start_* is set to the start of that period. In the same way, for the cells that did not divide during the period of interest, *t_end_* is set to the end of that period. ${f}_{t_{start}}$ and ${f}_{t_{end}}$ are the frames corresponding to *t_start_* and *t_end_*, respectively. We did not estimate ${r}_{cell}$ of the cells that were born before the start of the experiments (high-nutrient period). The number of cells analyzed in each microcolony were shown (Table S2).

For the computation of *r_subtree_,* we first estimated a total cell area of a subtree of interest (we call this parameter as *S*). *S* at time *t* in a subtree is formulated as follows.


(4) \begin{equation*} {\displaystyle \begin{array}{c}S(t)={\sum}_{i=1}^n{s}_i(t)\end{array}} \end{equation*}

Where, ${s}_i(t)$ is cell size (μm^2^) of a progeny cell existing at time *t* in a targeted subtree. The size of a subtree is also thought to grow exponentially as $S(t)={S}_0{e}^{r_{subtree}t}$, where *r_subtree_* is the size increase rate constant and ${S}_0$ is the initial size of a subtree. Therefore, the size increase rate ${r}_{subtree}(t)$ at time *t* in a given time frame $\Delta t$ could be approximated as


(5) \begin{equation*} {r}_{subtree}(t)=\frac{1}{\Delta t}\bullet \log \left(\frac{S\left(t+\Delta t\right)}{S(t)}\right) \end{equation*}

We further calculated the moving mean of ${r}_{subtree}(t)$ within $\Delta f$ (frames) of timepoint *t* as follows.


(6) \begin{equation*} \overline{r_{subtree}(t)}\ =\frac{1}{\Delta f}\#\sum_i{r}_{subtree}\left(t+i\Delta{t}^{\prime}\right)\#, for-\frac{\Delta f-1}{2}\le i\le \frac{\Delta f-1}{2} \end{equation*}

This moving mean $\overline{r_{subtree}(t)}$ was defined as the size increase rate of a subtree at time *t.* Then, ${r}_{subtree}$, average $\overline{r_{subtree}(t)}$ in a given period of time, from *t* = *t_start_* to *t = t_end_* as follows.


(7) \begin{equation*} {r}_{subtree}\ =\frac{1}{f_{t_{end}}-{f}_{t_{start}}}\#{\sum}_{t={t}_{start}}^{t={t}_{end}}\overline{r_{subtree}(t)} \end{equation*}

To calculate the size increase rate of a lineage (*r_lineage_*), we first made a list of ancestors of each cell present at the end of the low-nutrient period, by tracing back the lineage to the start of the experiments. The list of ancestral cells for each lineage starting from *t* = *t_start_* to *t = t_end_* is written as $\left[{a}_0,{a}_1,{a}_2,\dots{a}_n\right]$ (Fig. S2B). Since *r_lineage_* at time *t* is equivalent to the size increase rate of a single ancestral cell existing at that time, it can be described as ${r}_{lineage}(t)={r}_{cell}\left(t,{a}_k\right)$, where ${r}_{cell}\left(t,{a}_k\right)$ represents a size increase rate at time *t* of an ancestral cell ${a}_k$. Given the exponential growth in cell size (Fig. S1), ${r}_{cell}\left(t,{a}_k\right)$ is described as follows.


(8) \begin{equation*} {r}_{lineage}(t)={r}_{cell}\left(t,{a}_k\right)=\log \left(\frac{s_{a_k}\left(t+\Delta t\right)}{s_{a_k}(t)}\right)\bullet \frac{1}{\Delta t} \end{equation*}

where ${s}_{a_k}$ is the cell size of the ancestral cell ${a}_k$ at a given timepoint which exists at time *t*. Then we calculated moving mean for ${r}_{cell}\left(t,{a}_k\right)$ as follows.


(9) \begin{equation*} \overline{r_{cell}\left(t,{a}_k\right)}\ {\displaystyle \begin{array}{c}=\frac{1}{\Delta f}\#\sum_i{r}_{cell}\left(t+i\Delta{t}^{\prime },{a}_k\right)\#, for-\frac{\Delta f-1}{2}\le i\le \frac{\Delta f-1}{2}\ \end{array}} \end{equation*}

We calculated the average of this moving mean of size increase rate from *t* = *t_start_* to *t = t_end_* as


(10) \begin{align*} {r}_{lineage}=&\ \frac{1}{f_{t_{end}}-{f}_{t_{start}}}\left({\sum}_{t={t}_{start}}^{t={t}_1}\overline{r_{cell}\left(t,{a}_0\right)}+{\sum}_{t={t}_1}^{t={t}_2}\overline{r_{cell}\left(t,{a}_1\right)} \right. \nonumber \\ & \left. +\cdots +{\sum}_{t={t}_n}^{t={t}_{end}}\overline{r_{cell}\left(t,{a}_n\right)}\right) \end{align*}

where, $t={t}_1,{t}_2,{t}_3,\dots, {t}_n$ corresponds to the birth time of the ancestral cells ${a}_1,{a}_2,{a}_3,\dots, {a}_n$ (Fig. S2B). As ${r}_{cell}\left(t,{a}_k\right)$ is always a size increase rate of a single cell ${a}_k$, changes in the cell size from ${a}_k$ to ${a}_{k+1}$ (cell division event) are not included here.

We use *Δt´* = 10 min, *Δt* = 30 min, and *Δf* = 3 frames in the high-nutrient and recovery periods, and *Δt´* = 40 min, *Δt* = 400 min, and *Δf* = 9 frames in the low-nutrient period. For calculation of the moving mean near the start or end of periods, where existing frames before and after a given timepoint are usually less than *Δf*, we shorten *Δf* and include only existing frames for the calculation.

### Analysis for the effects of genealogical relatedness and spatial proximity

Effects of genealogy and spatial proximity on the regrowth in the recovery period were analyzed as previously described [26]. We focused on the *p_recovery_* of starter cells as a phenotype of interest (*X*) and used the spatial and genealogical information at *t =* 4680 min in each microcolony. For estimating the effect of genealogy, we compared *p_recovery_* within a group of three cells: a cell of interest, its closest relative, and an “equidistant” cell. The closest relative is the sister cell of the cell of interest, and the equidistant cell is a neighbor of the closest relative and has a distance from the cell of interest (*d_ED_*) that is equivalent to that between the cell of interest and its closest relative (*d_CR_*). The difference in the phenotype among those three cells were computed as follows.


$$ {\delta}_{CR}=\left|{X}_I-{X}_{CR}\right| $$

$$ {\delta}_{ED}=\left|{X}_I-{X}_{ED}\right| $$

Then the difference between those two parameters, *δ_ED_ – δ_CR_*, is used as a metric for the similarity in *p_recovery_* between the closest relatives compared to that between the cell of interest and the equidistant cell.

For estimating the effect of spatial proximity, we compared *p_recovery_* within a group of three cells: a cell of interest, its closest neighbor, and the “equally-related” cell. The closest neighbor is defined as a cell that is directly adjacent (within 3/4 cell width) to the cell of interest and whose center-to-center distance to the cell of interest is the closest. The equally-related cell has the same genealogical relatedness to the cell of interest as the closest neighbor does, but is most distant from the cell of interest (*d_ER_*). The difference in the phenotype among those three cells were computed as follows.


$$ {\delta}_{NB}=\left|{X}_I-{X}_{NB}\right| $$

$$ {\delta}_{ER}=\left|{X}_I-{X}_{ER}\right| $$

The difference between the two parameters, *δ_ER_ – δ_NB_*, represents the similarity in *p_recovery_* between the neighbors compared to that between the cell of interest and the equally-related cell.

## Results

### Diversification of cellular growth dynamics during the low-nutrient period in a clonal population

**Figure 1:**
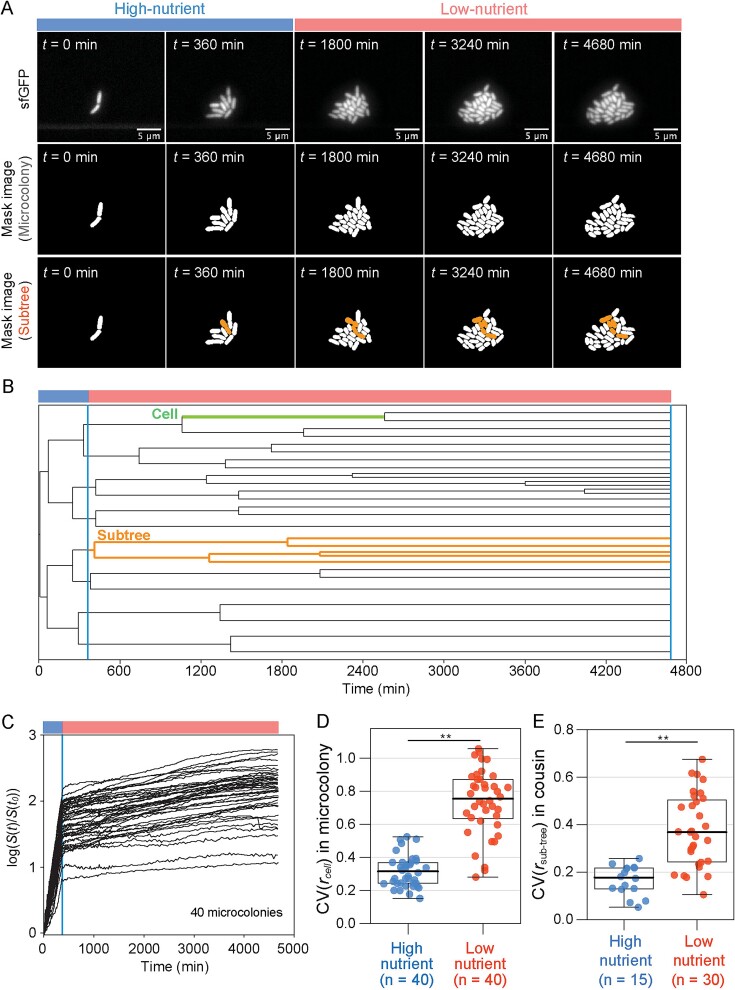
**Heterogeneous growth dynamics in the low-nutrient period.** (A) Time-lapse microscopic images of an *E. coli* microcolony from high-nutrient (*t* = 0–360 min) to low-nutrient (*t* = 360–4680 min) periods. We obtained mask images from sfGFP fluorescence images to quantify cell area (μm^2^). A subpopulation (designated subtree) derived from a single cell present at *t* = 360 min is indicated in the bottom images. (B) A representative lineage tree of a single microcolony. The tree is created by using time-lapse data of (A) and derived from a single cell at the start of the high-nutrient period. In a context of the tree, a cell is defined as a branch from its birth to division (green line). A subtree is a subpopulation derived from a cell at a given time point. The subtree shown in (A) is illustrated in orange lines. Vertical lines correspond to the timing when nutritional conditions were switched. (C) Growth curves of all 40 microcolonies obtained from two individual experiments. The size (i.e. area occupied by cells) of each microcolony *S*(*t*) compared to its initial size *S*(*t_0_*) is plotted over time on a log scale. A blue vertical line shows the timing of the shift from high- to low-nutrient periods (*t* = 360 min). (D) Coefficient of variation (CV) in size increase rate of cells within a microcolony. The average size increase rate of each cell (*r_cell_*) was calculated from its birth to division (or start or end of each period if birth or division events did not occur), and then the CV was calculated in a microcolony for high-nutrient and -poor periods separately. (E) CV in size increase rate within cousin subtrees. As the criteria in Fig. S6, 15 and 30 sets of cousin subtrees from 40 microcolonies were analyzed for high- and low-nutrient periods, respectively. Asterisks indicate the statistical significance level of Wilcoxon rank-sum test (**, *P* < . 01).

To investigate cellular growth dynamics in a clonal microbial population, we fabricated a custom microfluidic device with a membrane-covered microchamber array [27], for tracking growth of *E. coli* microcolonies at the single-cell resolution (Fig. S3) [28, 29]. An important feature of this device is the separation of cells from the flow channel with the medium by a semipermeable membrane, across which the medium diffuses but cells do not. This allows us to observe live responses of dividing cells under constant nutrient inflow as well as under instantaneously switching culture conditions. *E. coli* cells were first supplied with M63 minimal medium with 0.02% glucose to attain rapid growth for 6 hours (hereafter designated “high-nutrient period”). Then, we switched the medium to one without glucose and incubated the cells for 72 hours (hereafter designated as the “low-nutrient period”). Using 40 microcolonies observed from two independent experiments, we found that population growth (i.e. microcolony area occupied by cells) immediately stagnated but did not completely stop by the nutrient downshift (Movie S1 and Fig. 1A-C). Growth of individual cell lineages in a microcolony also abruptly slowed down but continued slowly, with obvious difference among lineages (Fig. S4A), suggesting heterogeneous responses during the low-nutrient period. To evaluate the heterogeneity among cells, we quantified cell sizes (i.e. cell area, denoted as *s*(*t*)) and calculated the rate of size increase from cell birth to division (denoted as *r_cell_*, average of log(*s*(*t + Δt*)*/s*(*t*))/*Δt*, see *Methods*). We found that the coefficients of variation (CV) of *r_cell_* in a microcolony were significantly higher in the low-nutrient than those in the high-nutrient period (Fig. 1D and Fig. S4B). Similarly, CV of generation time (i.e. the duration from birth to division) of individual cells in a microcolony were also higher in the low-nutrient period (Fig. S5A and B). One possible explanation for the higher growth heterogeneity in the low-nutrient period could be larger phenotypic variation due to a decrease of genealogical relatedness among cells over time, regardless of nutrient conditions [26, 30, 31]. Indeed, the average coefficient of relationship between cells (CR, defined as 2^–n^, where *n* stands for the generations to the closest common ancestor of two cells) in a microcolony was significantly higher in the high-nutrient period than that in the low-nutrient period, indicating the decrease of genealogical relatedness (Fig. S5C). If this is the reason, one would assume the CV of *r_cell_* becoming higher in the low-nutrient period, irrespective of the nutrient downshift. To exclude this effect, we analyzed growth of subtrees, which were subpopulations derived from single cells present at the beginning of high-nutrient period (*t* = 120 min) or at the start of the low-nutrient period (*t* = 360 min) in a microcolony (Fig. 1A and B, and Fig. S6), and compared growth of cousin subtrees, where CR among subtrees is always 0.25 (Fig. S6). The size of each subtree (denoted as *S*(*t*), orange area in Fig. 1A) was quantified, and its increase rate (*r_subtree_*, average of log(*S*(*t + Δt*)*/S*(*t*))/*Δt*, see *Methods*) was calculated in an equivalent time interval with respect to the generation time of each period (*i.e.* twice the median generation time of each period, 240 min for high-nutrient and 3160 min for low-nutrient) (Fig. S5A). This showed that the CV of *r_subtree_* for cousin subtrees was significantly higher in the low-nutrient than that in the high-nutrient period (Fig. 1E). These results thus demonstrate that *E. coli* cells abruptly decrease but do not completely stop their growth in the low-nutrient period, where the variation in cellular growth becomes larger than that in the high-nutrient period, irrespective of genealogical relatedness among cells.

### Diverse lag times after nutrient upshift are related to cellular growth histories

**Figure 2:**
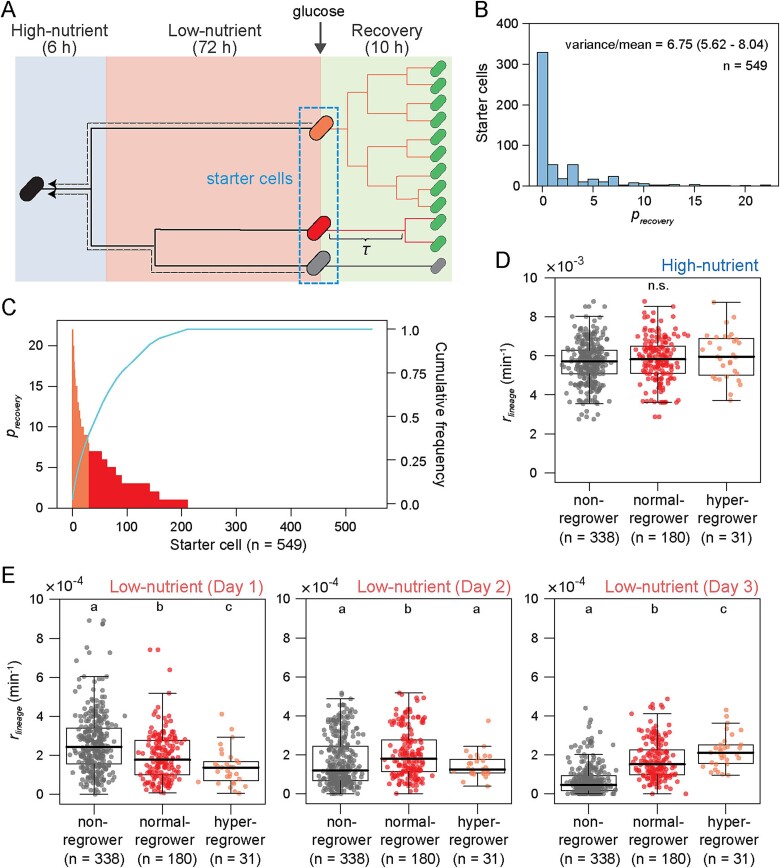
**Heterogeneous reproduction and growth history.** (A) A schematic illustration of the analysis. We observed regrowth of starter cells present at the beginning of the recovery period (encircled by dotted line) and analyzed their lag times *τ* (time to first cell division) and progeny numbers in the recovery period (*p*_recovery_). Starter cells were categorized into three groups based on their regrowth capacity: Non-regrower (gray), *p*_recovery_ = 0; normal-regrower (red), 0 < *p*_recovery_ ≤ 7; hyper-regrower (orange), *p*_recovery_ > 7. Growth histories of starter cells were analyzed retrospectively (dotted arrows), where we traced back the growth of each starter cell from the end of the low-nutrient period and estimated “past” size increase rates (*r_lineage_*) in high- and low-nutrient periods. (B) Distribution of *p*_recovery_ in starter cells. A total of 549 starter cells (in 28 microcolonies from one of the two independent experiments) were used for the analysis. (C) Sorting of starter cells in descending order of *p*_recovery_. The same data as in (B) was used. A curve indicates the cumulative proportion of total progeny. Hyper-regrowers and normal-regrowers are indicated in orange and red bars, respectively. Note that progeny cells from hyper-regrowers cover almost 50% of total progeny. (D) and (E) Comparison of growth histories among starter cells. Starter cells are categorized into the three groups, and their past *r_lineage_* in the high-nutrient period (*t* = 0–360 min) (D), Day 1 (*t* = 360–1800 min), Day 2 (*t* = 1800–3240 min), and Day 3 (*t* = 3240–4680 min) of the low-nutrient period (E) are separately plotted. The same data as in (B) was used. Median is shown as a thick horizontal line in each box. Alphabets show statistical significance groups according to Kruskal Wallis test followed by Steel-Dwass post hoc test (*P* < .05). n.s. means that no significantly different pairs exist.

After 3 days of the low-nutrient condition, we resupplied fresh M63 with 0.02% glucose to the device and observed growth dynamics for a further 10 hours (designated as “recovery period”) (Fig. 2A). We found substantial variation in lag time *τ* (time to first cell division) of individual cells present at the beginning of the recovery period (designated as “starter cells”) (Fig. S7A and B), and ~40% of the starter cells could regrow (dividing at least once in the recovery period) with comparable rates of size increase as those in the high-nutrient period (Fig. S7C). There was a strong positive correlation between the reciprocal of lag time (1/*τ*) and the number of progeny cells produced by each starter cell (*p_recovery_*) (Fig. S7D). The experimentally observed distribution of *p_recovery_* showed a peak at 0 with a long tail and a quite large variance-to-mean ratio (Fig. 2B and Fig. S8A). This suggests that the descendants produced in the recovery period were biased to specific starter cell origins. Indeed, 50% of the descendants were derived from the top ≈ 10% of the starter cells (Fig. 2C and Fig. S8B), supporting the hypothesis that a small fraction of starter cells largely contributed to the production of progeny cells in the recovery period. We designated this top 10% highly reproductive starter cells as “hyper-regrowers” (*p_recovery_* > 7), other reproductive starter cells as “normal-regrowers” (0 < *p_recovery_* ≤ 7), and undivided starter cells as “non-regrowers” (*p_recovery_* = 0), which refer to no growth within 10 hours of the recovery period but do not exclude the possibility of regrowth after longer lag time.

We investigated how such biased reproduction emerged among starter cells. To explore the interrelation between reproduction and growth history of starter cells, we retrospectively traced cell lineages (Fig. 2A, dotted arrows), and quantified their rates of size increase (*r_lineage_*) and the number of generations (i.e. the number of division events). Neither *r_lineage_* nor the number of generations in the high-nutrient period were statistically significantly different among hyper-, normal-, and non-regrowers (Fig. 2D, Fig. S9A, and Fig. S10A), while the number of generations in the low-nutrient period was significantly smaller in hyper-regrowers than others (Fig. S9A). Notably, *r_lineage_* among the three groups of regrowers in the low-nutrient period were substantially different and changed over time (Fig. 2E and Fig. S10A). Hyper-regrowers exhibited lower *r_lineage_* at Day 1 but higher at Day 3 than other regrowers, while it was maintained at constant rates around 1–2 × 10^4^ (min^−1^). In contrast, *r_lineage_* of normal- and non-regrowers gradually decreased over 3 days and were eventually lower than that of hyper-regrowers at Day 3. While absolute values of *r_lineage_* were slightly different between the two independent experiments, these qualitative trends were highly reproducible (Fig. 2E and Fig. S10A). In addition, the trends were consistently observed when the threshold of hyper-regrowers was changed to the top 5% or 3% (Fig. S11A and B). These suggest that high reproduction in the recovery period depends on the particular growth dynamics in which cells immediately suppressed but feebly maintained their growth during the low-nutrient period.

We assumed that physiological differences among cells at the switching from high- to low-nutrient periods lead to subsequent heterogenous growth responses and reproduction. A possible mechanism governing such differences could be different cell-cycle phases of cells present at the nutrient downshift, as many physiological traits should change according to the cell cycle [32–34]. To estimate cell-cycle phases when cells just entered the low-nutrient period (*t* = 360 min), we traced back a lineage of each starter cell and calculated the time from the last cell division in the high-nutrient period to the start of the low-nutrient period (*τ*_start_) (Fig. S12). However, we found no significant effect of τ_start_ on the reproductivity in the recovery period (Fig. S12D). The difference in τ_start_ affected the size increase rate only at Day 1 but not at later days in the low-nutrient period (Fig. S12E), suggesting that the difference in cell-cycle phases at the transition point has little impact on subsequent growth dynamics and reproduction.

### Growth suppression is key for reproduction even after shorter starvation

**Figure 3:**
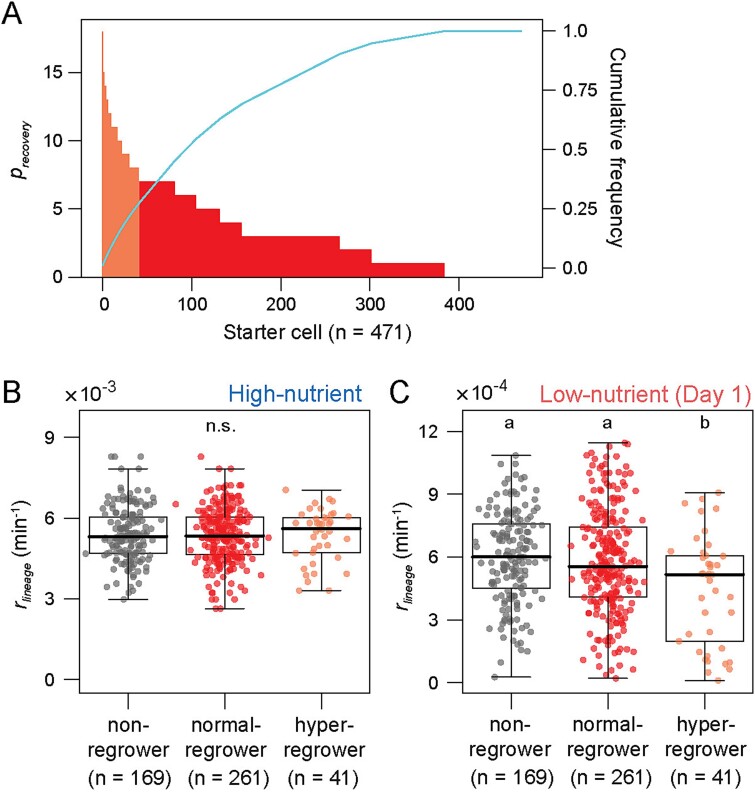
**History-dependent growth resumption after the shorter low-nutrient period.** (A) Reproduction of starter cells (*p*_recovery_) after 1 day of the low-nutrient period. A total of 471 starter cells (in 25 microcolonies from one of the two independent experiments) were sorted in descending order of *p*_recovery_. All configurations are same as Fig. 2C. (B) and (C) Comparison of the past *r_lineage_* in three types of starter cells. The *r_lineage_* in the high-nutrient (*t* = 0–360 min) (B) and the low-nutrient (*t* = 360–1800 min) (C) periods among the 471 lineages are displayed. Median is shown as thick black line in each box. Alphabets show statistical significance groups according to Kruskal Wallis test followed by Steel-Dwass post hoc test (*P* < .05). n.s. means that no significantly different pairs exist.

Given that the three groups of regrowers exhibited characteristic growth histories, we examined whether those histories were still observed if glucose was resupplied earlier. We performed again time-lapse microscopy experiments with 1 day instead of 3 days of the low-nutrient period and traced lineages of starter cells (Movie S2). This 1-day condition resulted in ~65% of the starter cells regrowing, higher than that after 3 days of the low-nutrient period (i.e. 40%), but the proportion of hyper-regrowers (*p_recovery_* > 7) remained consistent at ~10% in both cases (Fig. 2C, Fig. 3A, Fig. S8B, and Fig. S10B). The increase in the total number of regrowing cells was thus due to an increase in the number of normal-regrowers (0 < *p_recovery_* ≤ 7). Both the number of generations and *r_lineage_* during 1 day of the low-nutrient period were significantly lower in hyper-regrowers than others, while no statistical difference was found in those parameters during the high-nutrient period (Fig. 3B and C, Fig. S9B, and Fig. S10C). This trend was still observed with the different thresholds of hyper-regrowers (Fig. S11C and D). These results confirm that cellular reproduction in the recovery period depends on the growth history in the low-nutrient period but not in the high-nutrient period, and suggest that immediate suppression of cell growth after the nutrient downshift promotes starter cells to become hyper-regrowers. Moreover, while normal-regrowers exhibited lower *r_lineage_* than non-regrowers at Day 1 in 3 days of the low-nutrient period (Fig. 2E), those were indistinguishable in the 1-day condition (Fig. 3C), suggesting that cells which cannot regrow after the 3-day condition still have a potential of regrowth after the shorter condition.

### Reproduction is dependent on cell lineage but not on spatial proximity

**Figure 4:**
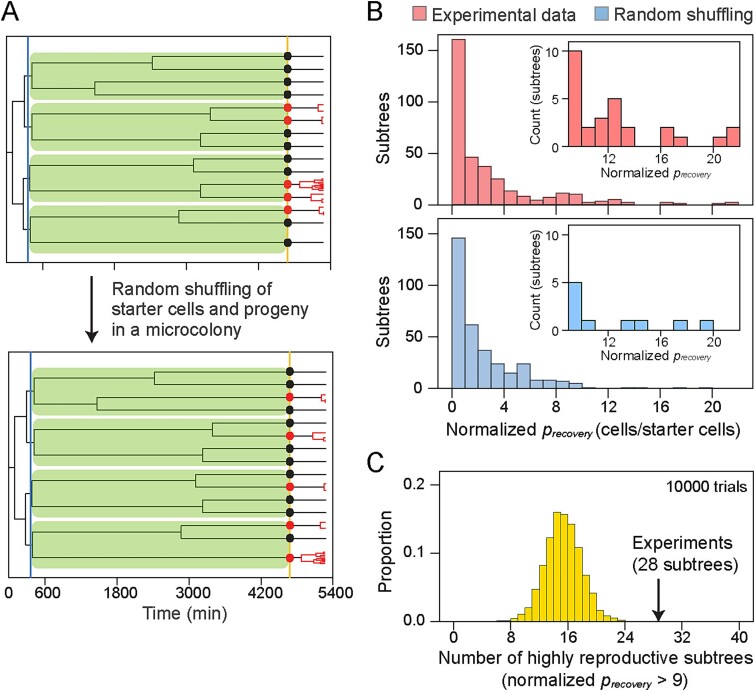
**Inheritance of the regrowth capability.** (A) A schematic illustration of random shuffling of starter cells and their progeny. Subtrees derived from single cells presented at the onset of low-nutrient were shaded. While keeping the structure of subtrees, all the starter cells (black and red circles) with their progeny are randomly shuffled within each microcolony. (B) Distributions of normalized *p_recovery_* of subtrees before and after random shuffling. The random shuffling was performed in each microcolony, and shuffled data from 40 microcolonies were merged. A representative distribution of normalized *p_recovery_* after the shuffling (blue bars) is shown with the experimental data (red bars). Each inset is an enlarged view of the higher part of the distribution (i.e. normalized *p_recovery_* > 9, which is 1.5 times of quartile range from the 3/4 quantile in experimental data). (C) Distribution of the number of highly reproductive subtrees (normalized *p_recovery_* > 9) in random shuffling data (10 000 trials). A black arrow indicates the number of highly reproductive subtrees observed in experiments.

Given that reproduction of starter cells is related to their growth histories in the low-nutrient period, we hypothesized that starter cells in the same subtree exhibit similar reproduction since they share part of their lineages. In that case, highly reproductive starter cells could be clustered in specific subtrees rather than randomly distributed in a population. To test this hypothesis, we calculated the normalized *p_recovery_* for subtrees (i.e. the number of progeny cells divided by the number of starter cells in a given subtree) using experimental data of the 3-day low-nutrient condition, and compared them with those generated by random shuffling of starter cells and their progeny in a microcolony (Fig. 4A). Based on the experimental data, highly reproductive subtrees were here defined as outliers that exhibit normalized *p_recovery_* ≥ 9 (more than 1.5 times interquartile range above the third quantile data). We repeated shuffling 10 000 times and found that the number of highly reproductive subtrees in the shuffled data was never larger than that observed in the experiments (Fig. 4B and C). This suggests that highly reproductive starter cells are derived from specific subtrees. In addition, there was a positive, albeit not so strong, correlation between genealogy and reproductive success in pairs of starter cells: the higher the coefficient of relationship was, the higher the correlation coefficient of *p_recovery_* became (Fig. S13).

As the closely related cells usually neighbor spatially each other [26], one may assume that the observed significant correlation of *p_recovery_* between genealogically related cells could be due to local effects caused by their spatial proximity (e.g. cell–cell interaction or signal molecules released from cells) rather than cellular genealogy. To examine the possibility, we distinguished the genealogical relatedness from the spatial proximity, and analyzed their effects separately on reproduction of starter cells. We computed dissimilarity scores in *p_recovery_* between pairs of spatially proximal (*δ_NB_*) or genealogically proximal (*δ_CR_*) starter cells, and found the significant effect of the genealogical relatedness but not the spatial proximity of starter cells on *p_recovery_* (Fig. S14). Taken together, we conclude that heterogeneity in growth resumption among starter cells is derived from different cellular physiology among ancestral cells in the low-nutrient period rather than stochastic emergence of the variation by resupplying glucose to starter cells nor spatial proximity.

### Fatty acid metabolism is responsible for growth dynamics in the low-nutrient period and subsequent growth resumption

**Figure 5:**
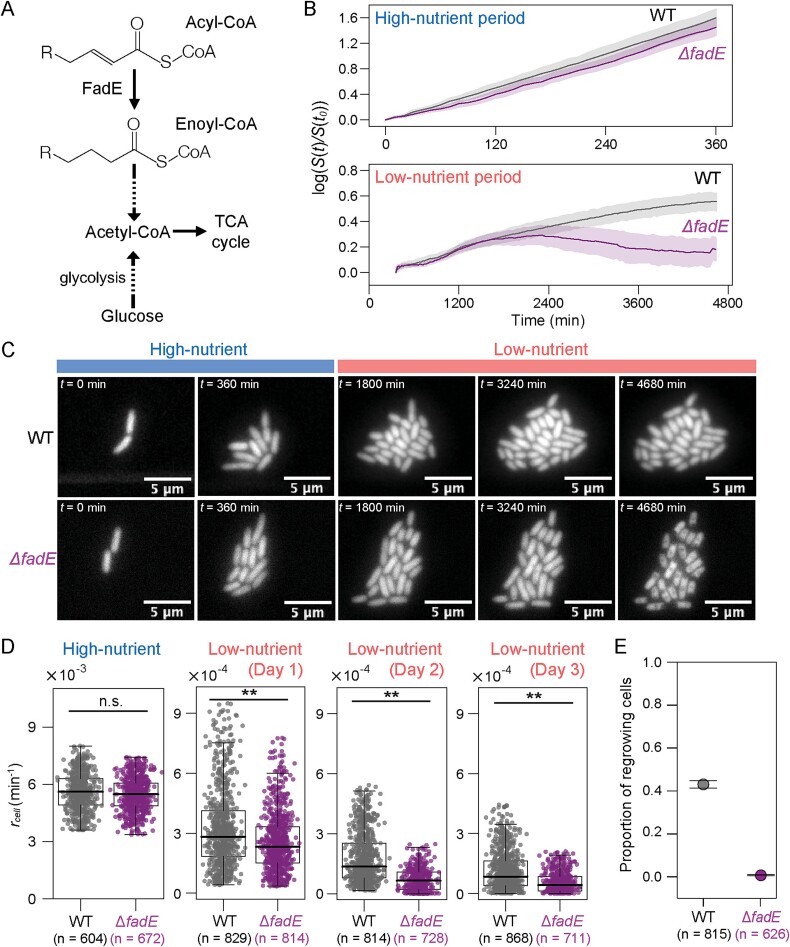
**Effect of the acyl-CoA dehydrogenase (FadE) disruption on cell growth and subsequent reproduction in the recovery period.** (A) Fatty acid β-oxidation pathway in *E. coli.* FadE is the first enzyme to break down a long-chain acyl-CoA to acetyl-CoA. (B) Growth curves of *E. coli* WT (black) and Δ*fadE* (purple) microcolonies. The size of the microcolony *S*(*t*) compared to its initial size *S*(*t_0_*) is plotted over time on a log scale. Here we set *t_0_* to the beginning of each period (high-nutrient, *t* = 0 min; low-nutrient, *t =* 360 min). Average growth curves are indicated solid lines, and their 95% bootstrap confidence intervals are shaded. (C) Time-lapse fluorescence images of *E. coli* microcolonies from high-nutrient (*t* ≤ 360 min) to low-nutrient (*t* ≥ 360 min) periods in WT and *ΔfadE* strains. (D) Comparison of size increase rates of cells (*r_cell_*) in high- and low-nutrient periods. *r_cell_* was calculated from its birth to division (or start or end of each period if birth or division events did not occur). Data from all 40 (WT) and 18 (Δ*fadE*) microcolonies are shown. Statistical significance levels estimated by Wilcoxon rank sum test are indicated (**, *P* < .01; n.s., not significant). (E) Proportion of starter cells that divided at least once in the recovery period. In total, 815 WT and 626 Δ*fadE* starter cells obtained from 40 and 18 microcolonies, respectively, were analyzed. Error bars indicate 95% bootstrap confidence intervals.

We investigated metabolic processes underlying cellular response in the low-nutrient period. Previous studies had suggested that degradation of cellular membrane lipids to acetyl-CoA by β-oxidation is an essential pathway to obtain carbon and energy sources in starved cells [19, 35]. Indeed, we recently showed that expression of the *fad* genes, encoding enzymes to degrade fatty acids via β-oxidation, is significantly upregulated when *E. coli* cells are under starvation conditions [36]. To test our hypothesis that the degradation of fatty acids through β-oxidation is responsible for cellular growth in the low-nutrient period, we used a *fadE* mutant lacking an acyl-CoA dehydrogenase in β-oxidation (Fig. 5A) and measured its cellular growth dynamics (Fig. 5B and C, and Movie S3). While no significant difference in rates of size increase between WT and the mutant cells was observed in the high-nutrient period, the mutant showed statistically significantly lower rates during the 3-day low-nutrient period (Fig. 5D). Retrospective analysis of cell lineages revealed that starter cells of the *fadE* mutant exhibited comparable growth phenotypes to normal-regrowers of WT at Day 1 in the low-nutrient period but then lower rates than regrowers of WT at Days 2 and 3 (Fig. S15). These results indicate that the β-oxidation process contributes to maintaining feeble cell growth in the low-nutrient period. We further found that few cells (only four of 626 starter cells) of the mutant subsequently resumed their growth in the recovery period (Fig. 5E), suggesting that the fatty acid β-oxidation cycle could play a crucial role in the adaptive cellular response in the low-nutrient period for future growth resumption with a new carbon source.

## Discussion

Most bacteria live in natural environments where nutrient supply fluctuates dynamically. Recent studies have revealed that, in a clonal bacterial population, not all but only a fraction of cells can resume their growth from days of starvation or a dormant state when new carbon sources are provided [7, 8, 37]. Yet, how such heterogeneous responses emerge in an isogenic population has not been well understood. We here discovered diversification of single-cell growth dynamics upon a shift from high- to low-nutrient conditions (Fig. 1) and subsequent diverse cellular responses for growth resumption when a new carbon source was resupplied (Fig. 2). Our previous work using *E. coli* populations shows distinct profiles of gene expression at different time points of stationary phase, where cell growth is almost arrested [36]. We thus assumed that many physiological traits could change during 3 days of the low-nutrient period. However, the future growth resumption depended on cellular physiological states developed in the beginning of the low-nutrient period, suggesting that the ability to regrow is determined in the beginning of the starvation while other cell functions or gene expressions might change over time. These results are in agreement with previous batch-based studies showing the presence of a long-term memory effect on the culture regrowth from starved or growth-arrested states [14, 16]. Our single-cell tracking analysis first demonstrated the presence of inherited phenotypic trait for future growth resumption from the nutrient limited condition, although mechanistic details of inheritance need to be further investigated as it is known that a small variation emerged in the ancestral cells upon nutrient changes could be amplified [38].

One may assume that the reason of such diversification of growth dynamics in the low-nutrient period and subsequent different regrowth capacities among cells could be differences in the cell cycle at the shift from high-nutrient to -poor periods. It is indeed known that various physiological traits, such as gene expressions and concentration of metabolites, fluctuate during the cell cycle [32–34]. However, our results showed no significant difference in the reproductive success among starter cells whose ancestor cells were in different cell-cycle phases at the timing of the nutrient downshift (Fig. S12). The difference in the cell cycle was related to the rate of size increase only at Day 1 (the first 24 hours) in the low-nutrient period but no longer in following stages. A previous study has reported that the cell-cycle state at the onset of salt stress affects the ability of cells to divide within a few hours [39]. Physiological heterogeneity derived from different cell-cycle phases at the onset of environmental change might affect the subsequent cellular growth for hours but not longer as this study tested.

Our results show a strong contribution of the fatty acid β-oxidation process to feeble growth in 3 days of low-nutrient condition and subsequent growth resumption in the recovery period. The role of the β-oxidation cycle for survival under carbon starvation has been widely reported across taxonomically distant microbial species [40–42]. Expression of the *fad* genes of *E. coli* for the fatty acid degradation is controlled by a transcriptional regulator FadR with an acyl-CoA-dependent manner: FadR binds to an operator region of *fad* genes and represses the transcription under high-nutrient conditions (i.e. absence of exogenous acyl-CoA), while acyl-CoA binding of FadR derepresses the transcription in the nutrient-limited conditions [43]. One may assume that the level of β-oxidation activity is a determinant of becoming either (hyper-)regrowers or non-regrowers. Our previous work shows that the *fadR* expression level does not change between late-exponential and stationary phases at the population level [36], suggesting that intracellular amount of acyl-CoA is more likely to be crucial for the formation of acyl-CoA-bound FadR. As the source of the acyl-CoA ligands is long-chain fatty acids [43], fluctuations of intracellular amount of fatty acids might affect β-oxidation activity and result in the growth heterogeneity. Another possible scenario is that β-oxidation activity is just one of the essential factors for the survival under low nutrients but its fluctuation does not trigger the cell differentiation. In this case, cellular variations of other intrinsic factors, such as ppGpp and stress resistance genes, might cause the growth heterogeneity. As an increase of ppGpp level generally inhibits cell growth [44], this might result in the growth suppression observed in hyper-regrowers. Further extended studies are needed to elucidate these molecular regulatory systems.

As the amount of carbohydrates readily available for cells becomes very low in our low-nutrient conditions, the degradation of intracellularly stored fatty acids via β-oxidation to yield acetyl-CoA is expected to be responsible for their survival and future growth resumption [36]. Another possibility is that the β-oxidation cycle works for utilization of extracellular fatty acids derived from other cells in the population. Previous studies have reported the importance of recycling carbon sources released from lysed or dead cells for population survival [45–47]. In our experimental condition, however, the contribution of the recycling activity might be considered to be small because cell-derived nutrients are more likely to be diffusing into the chamber through the semi-permeable membrane. In fact, there was no correlation between the rate of lysed cells and the average size increase rate of microcolonies in the low-nutrient period (Fig. S16). In addition, we found no spatial effects on cellular reproduction that could be due to extracellular molecules released from dead cells (Fig. S14).

Taken together, heterogeneity in growth resumption among cells is the consequence of their diverse growth history during the low-nutrient period, but not the result of stochastic emergence with nutrient resupply. Given that cellular physiology during the low-nutrient period depends on the metabolism of fatty acids, producing more progeny cells in early stages of the period (like non-regrowers) might lead to rapid consumption of internal energy, which would be unfavorable for survival and future regrowth with new carbon sources [16, 48]. Hyper-regrowers, in contrast, significantly suppressed their growth after nutrient downshift and then maintained feeble growth rates (less than 1/40 of those in the nutrient-rich period, Fig. 2), and could rapidly regrow and reproduce more progeny cells when nutrient resupplied. The latter cells would thus have a better adapted physiology to long-term survival and growth resumption. In contrast, if the starvation period is short, cells which could not immediately suppress their growth still have a potential to regrow with new carbon sources (like normal-regrowers). Whether rapid nutrient consumption or metabolic switching is costly should depend on the length of the nutrient-poor or starvation period. From these perspectives, the emergence of heterogeneous growth dynamics among clonal cells under nutrient-limited conditions would be one way to adapt microorganisms to the environment where nutritional conditions fluctuate irregularly.

## Supplementary Material

Takano_SupplementaryInfo_rev2_wrae257

MovieS1_wrae257

MovieS2_wrae257

MovieS3_wrae257

## Data Availability

All codes for data analysis and simulations can be found in the following GitHub repository: https://github.com/sotarotakano/Cell_lineage_Analyzer.
