# Supplementary material for: Diversification of single-cell growth dynamics under starvation influences subsequent reproduction in a clonal bacterial population
Source: ISME J. 2024 Dec 23;19(1):wrae257. doi: 10.1093/ismejo/wrae257 (PMC11773413; doi:10.1093/ismejo/wrae257)
Supplement: Takano_SupplementaryInfo_rev2_wrae257 [file takano_supplementaryinfo_rev2_wrae257.pdf]

Supplementary information for

Diversification of single-cell growth dynamics under starvation influences subsequent reproduction in a clonal bacterial population

Sotaro Takano, Miki Umetani, Hidenori Nakaoka, and Ryo Miyazaki

Ryo Miyazaki
Email: ryo.miyazaki@aist.go.jp

This PDF includes;
Figures S1 to S16
Table S1 and S2
Legends for Movies S1 to S3

Other supplementary materials for this manuscript include the following:
Movie S1 to S3

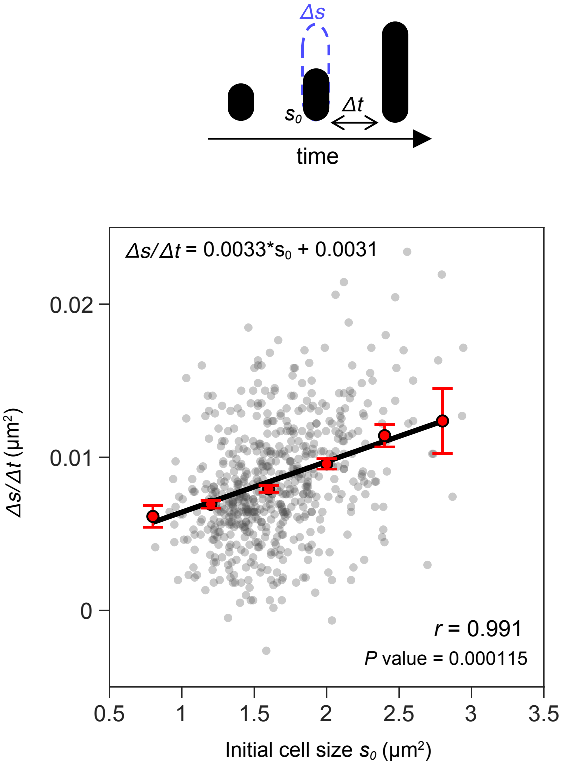

Fig. S1. Correlation between initial cell size (area) and increment of cell size at the single-cell level. Growth of 397 cells in 40 microcolonies born in the high-nutrient period was analyzed. Entire data are binned to 6 intervals (from 0.6 to 3 μm^2 in initial cell area), and mean $\Delta s/\Delta t$ values of the binned data are plotted as red circles. Error bars indicate standard error. Linear regression to the mean is indicated as a black line (the fitted parameters are shown with the formula in the upper left). Pearson correlation coefficient (r) for the binned data is indicated with P value.

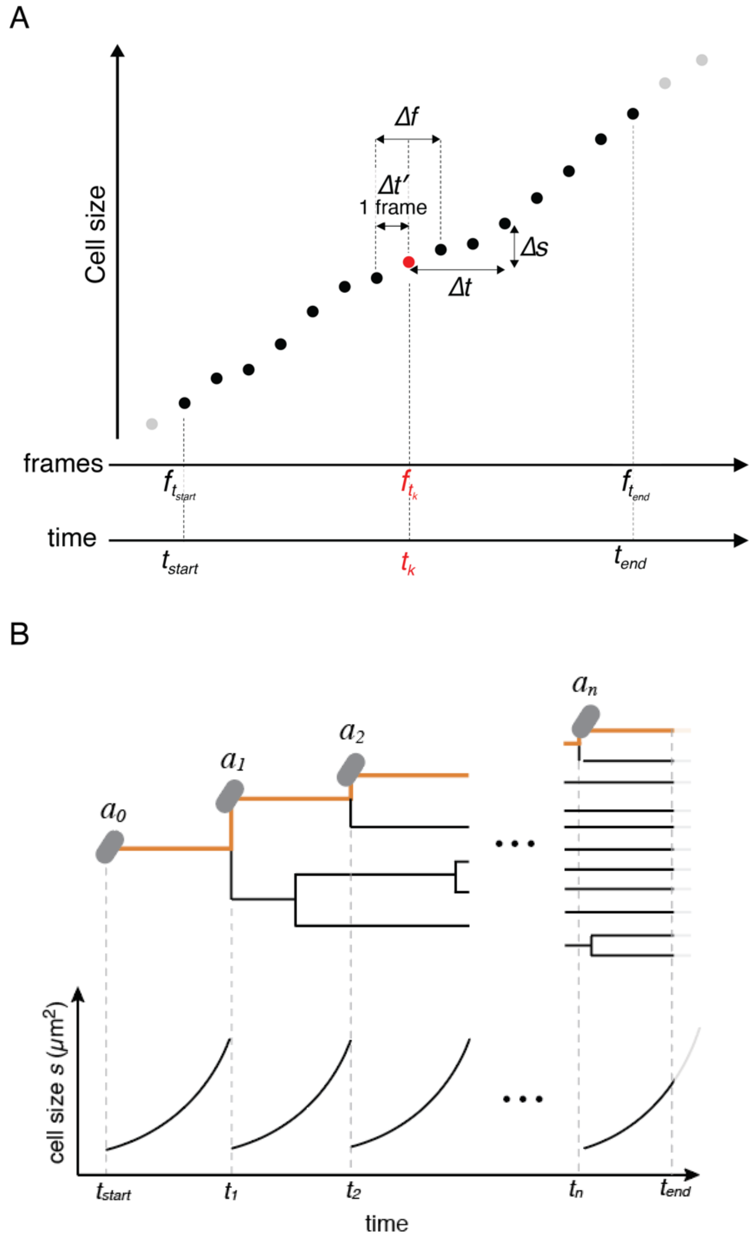

Fig. S2. Schematic illustrations of analysis of the size increase rate. (A) Parameters used for estimating the size increase rate. Each circle represents the cell size at each timepoint. Here, the analysis at $t = t_k$ (a red circle) in the high-nutrient period is shown ($\Delta t' = 10$ min, $\Delta t = 30$ min, and $\Delta f = 3$ frames). (B) A schema of lineage growth analysis. Here, we focus on the growth of an orange lineage from t_{start} to t_{end}. Parameters correspond to Methods.

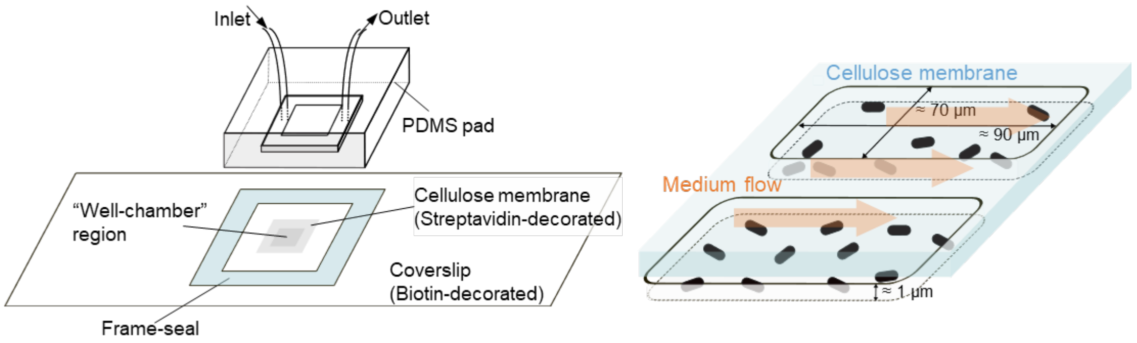

Fig. S3. Experimental setup for single-cell observations. Schematic of Well-chamber is illustrated. The microchambers (70 µm (l) ×90 µm (w)) were created directly on a glass coverslip by glass-etching (right panel). The depth of the microchambers was fabricated to be around 1 µm to allow bacterial cells to grow in a single layer (right panel). We sealed the microchambers by streptavidin-decorated cellulose membrane, which strongly binds to the biotin decorated on the surface of the coverslip. The PDMS pad was attached on top of it, and the culture medium was allowed to flow into the microchamber through the silicone tube attached to the PDMS pad.

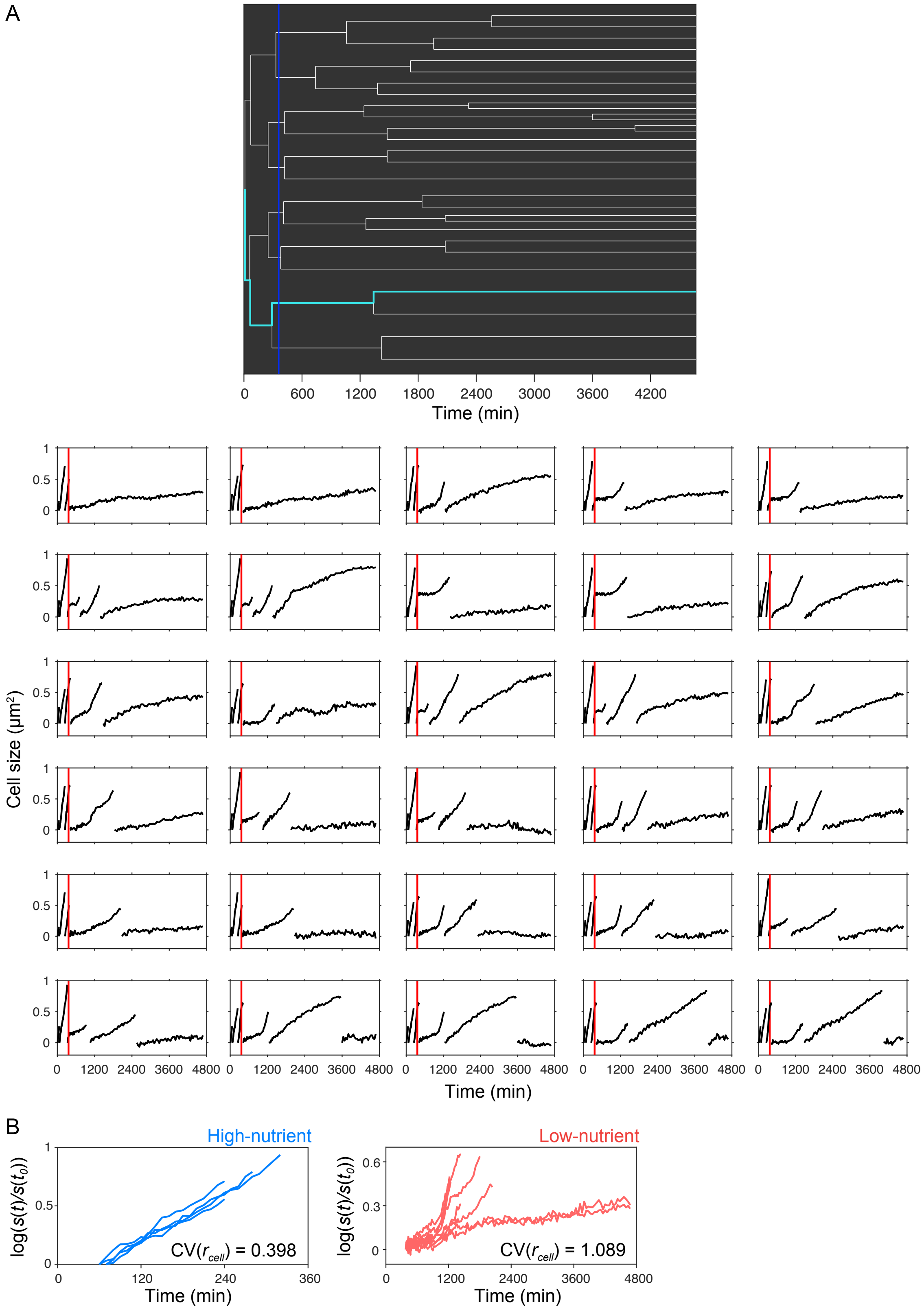

Fig. S4. Growth dynamics in a representative microcolony. (A) Growth curves of cell lineages. All 30 lineages derived from the single ancestral cell that existed at the start of the experiments were analyzed. A cyan line corresponds to a trace of one lineage. We

plotted temporal changes in cell size from the start (t = 0 min) of the high-nutrient period to the end of the low-nutrient period (t = 4680 min). Red vertical lines in bottom panels show the timing of the transition from the high-nutrient to low-nutrient period (t = 360 min). (B) Growth heterogeneity among cells. Typical growth curves of cells in high- and low-nutrient periods in the same microcolony. Cells born during t = 60-120 min (high-nutrient) and t = 360-480 min (low-nutrient) were selected, respectively. The size of each cell (s) compared to its initial size ($s(t_0)$) is plotted from its birth to division on a log scale. We set t_0 to the birth time of each cell, and plotted the log10-scaled relative changes in $s(t)$. Coefficient of variation (CV) in cell size increase rate among cells is indicated for each period.

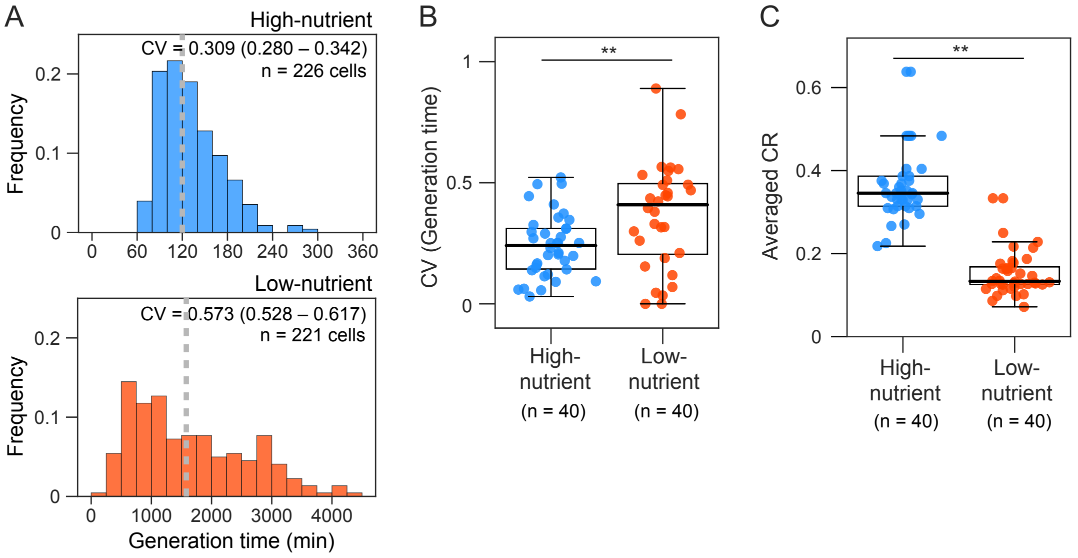

Fig. S5. Genealogical and growth properties of cells in high- and low-nutrient periods. (A) Distribution of generation time (*i.e.*, the duration from birth to division). We analyzed 226 (high-nutrient) and 221 (low-nutrient) cells in all 40 microcolonies, whose birth and division events were observed within each period. Cells which did not divide until the end of the period or whose birth event did not occur during the period were excluded. Median generation times (120 min and 1580 min for high- and low-nutrient periods, respectively) are indicated in vertical dotted lines. In each panel, coefficient of variation in generation time is displayed with 95% bootstrap confidence intervals. (B) CV of generation time of individual cells in a microcolony. Cells that were born and divided during each period (*i.e.*, cells born and divided until t = 360 min for the high-nutrient period, and those born after t = 360 min and divided until t = 4680 min for the low-nutrient period) were used for calculating generation time. CV in each microcolony (n=40) during high- and low-nutrient periods were calculated separately. Asterisks indicate the statistical significance level between two conditions ($P = 2.43 \times 10^{-4}$, Wilcoxon signed-rank test). (C) Average coefficient of relationship (CR) between cells in a microcolony. CR in each pair of cells in high- and low-nutrient periods was calculated and averaged at the microcolony level. All 40 microcolonies were analyzed. Asterisks indicate the statistical significance level between two conditions ($P = 3.56 \times 10^{-8}$, Wilcoxon signed-rank test).

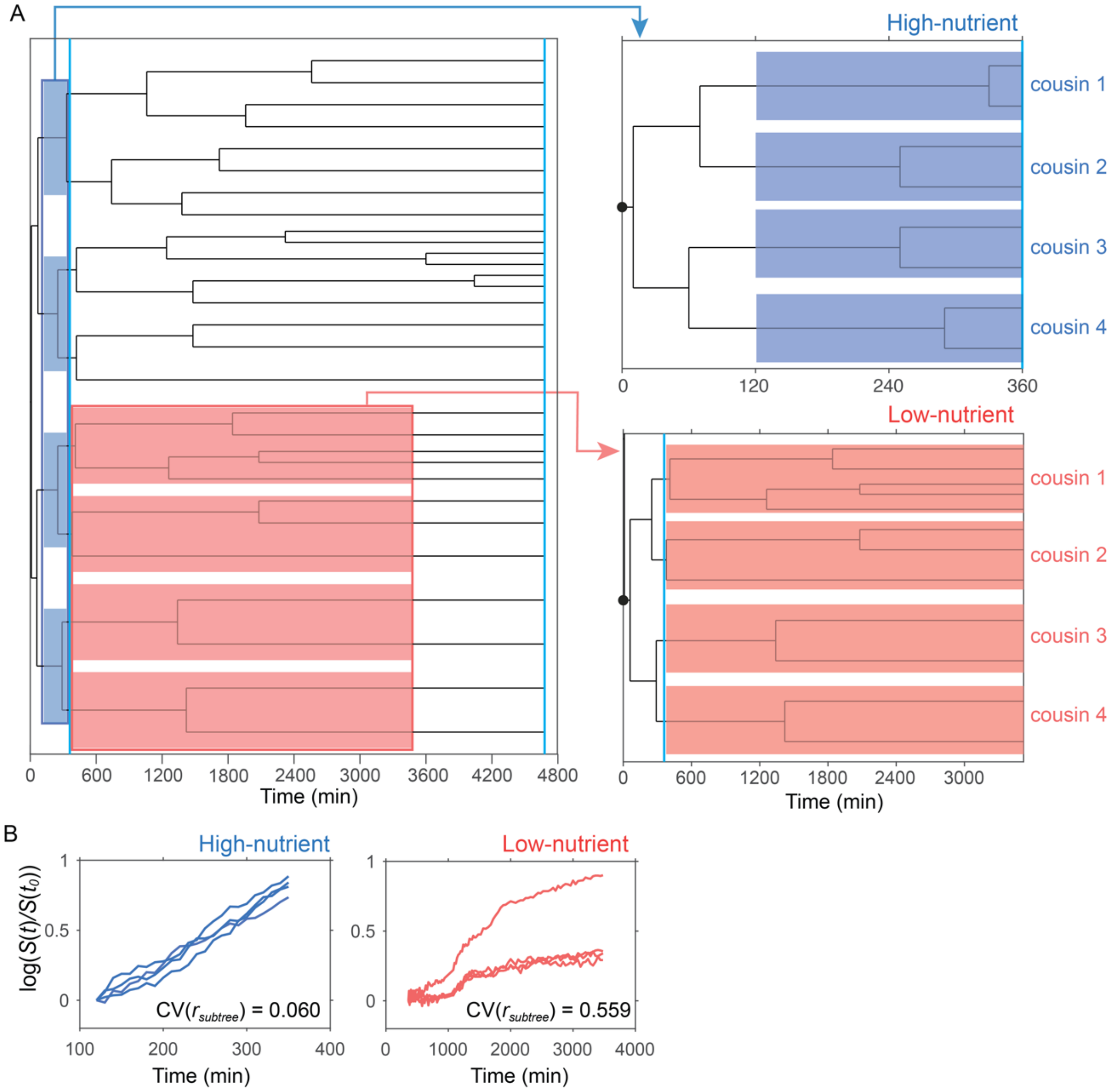

Fig. S6. Growth heterogeneity among cousin subtrees. (A) Typical sets of cousin subtrees in high-nutrient (shaded in blue) and low-nutrient periods (shaded in red). Right panels focus on those cousin subtrees with their grandparent cells (shown as black circles). Shaded regions correspond to timeframes used for the calculation of size increase rates: In the high-nutrient period, we selected cousin subtrees present at $t = 120$ min and analyzed their growth until $t = 360$ min, which corresponds to twice the median generation time (240 min), while in the low-nutrient period we selected cousin subtrees present at $t = 360$ min and analyzed their growth over 3160 min (twice the median generation time in the low-nutrient period). (B) Growth curves of cousin subtrees shown in (A). The size of each subtree (S) compared to its initial size ($S(t_0)$) is plotted over time on a log scale. We set t_0 to the starting point of the analysis in each period (high-nutrient: $t = 120$ min; low-nutrient: $t = 360$ min), and plotted the log10-scaled relative changes in $S(t)$. Coefficient of variation (CV) in size increase rate among cousin subtrees is indicated for each period.

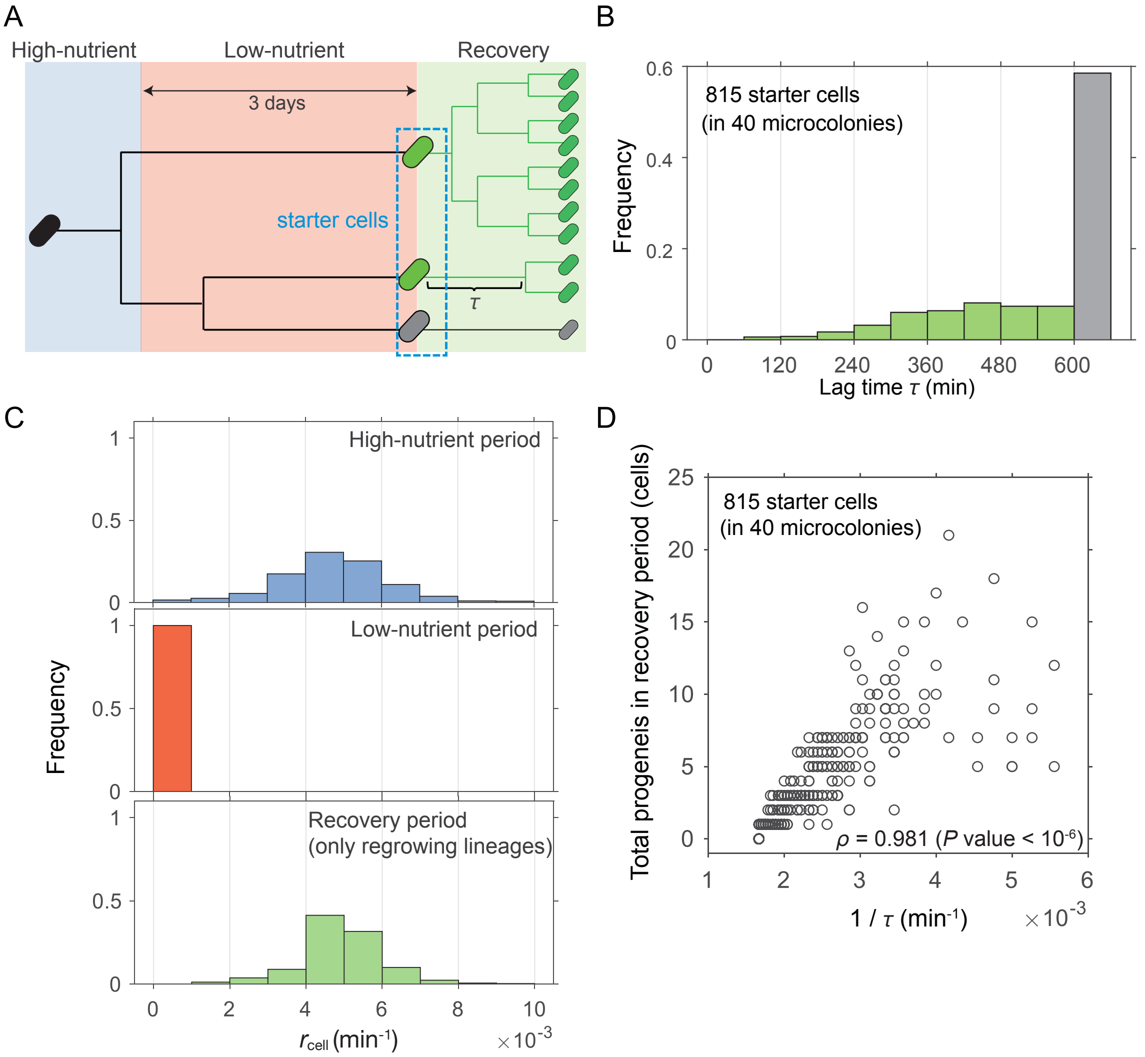

Fig. S7. Lag times and descendants of starter cells in the recovery period. (A) A schematic illustration of the analysis. Same as Fig. 2A. (B) Distribution of the lag time (τ). Lag times of starter cells that did not show the growth resumption in the recovery period were plotted as a gray bar. (C) Distributions of size increase rates at single cell level (r_{cell}) in high-nutrient, low-nutrient, and regrowth periods. We used 298 cells that were born and divided in the high-nutrient period and analyzed their growth data from their birth to division (blue). In the case of low-nutrient period, 1590 cells that were born and divided during the low-nutrient period were analyzed (red). In the case of recovery period, we used 356 cells that divided in the recovery period and analyzed their growth from 120 min before their first division to the time of the division event. (D) Correlation between reciprocal for lag time ($1/\tau$) and the number of progeny ($p_{recovery}$) of starter cells in the recovery period. Spearman's rank correlation coefficient (ρ) between those two parameters is indicated with P value.

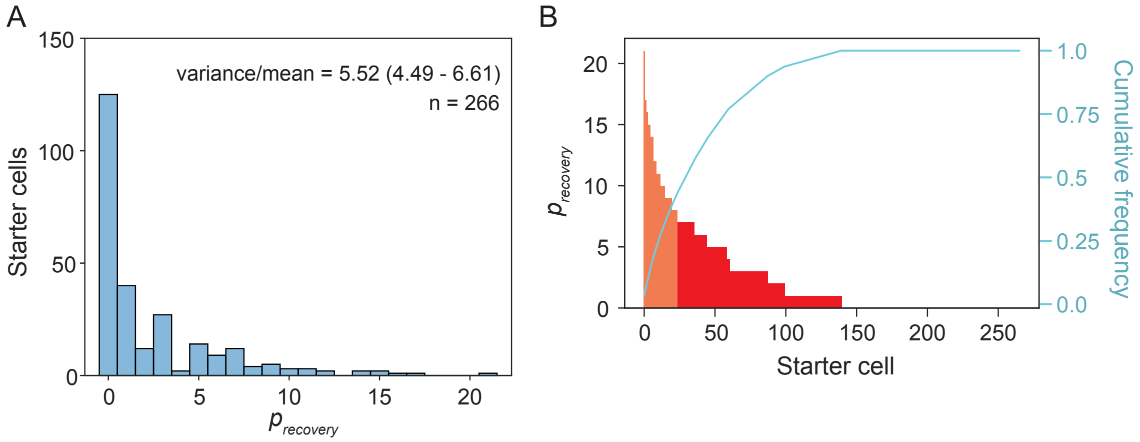

Fig. S8. Ability of starter cells to reproduce progeny cells. (A) Distribution of p_{recovery} in one of the two independent experiments. Data was from 266 starter cells in 12 microcolonies. Variance-to-mean ratio is displayed with 95% bootstrap confidence intervals. The result of the other experiment is shown in Fig. 2B. (B) Sorting of starter cells in descending order of p_{recovery} in one of two independent experiments. The same data as in (A) was used. Hyper-regrowers and normal-regrowers are indicated in orange and red bars, respectively. A cyan line indicates the cumulative proportion of total progeny. The result of the other experiment is shown in Fig. 2C.

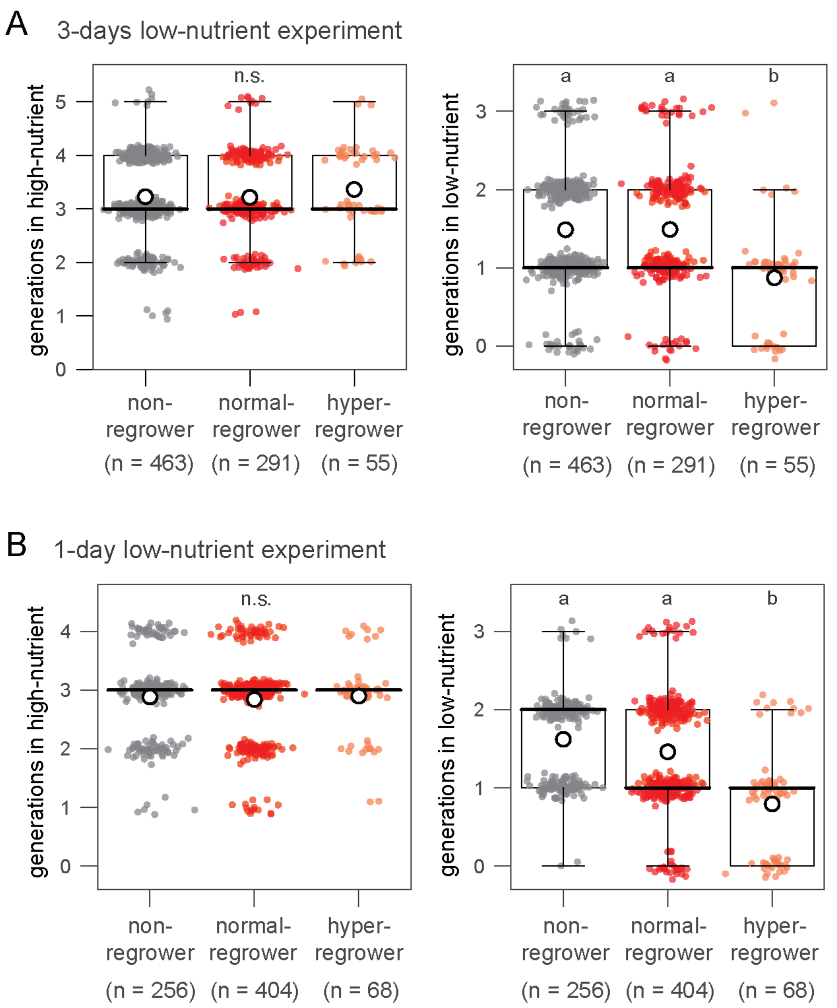

Fig. S9. Past generations of starter cells. The number of generations, corresponding to the number of division events, that each starter cell experienced in high- and low-nutrient periods are plotted. Results of the 3-days low-nutrient experiment (A) and the 1-day low-nutrient experiment (B) are shown. Data of the starter cells are merged data of the two independent experiments used in Fig. 2, Fig. 3, Fig. S8, and Fig. S10. Median and average values are shown as a thick black line and an open circle in each box, respectively. Alphabets show statistical significance groups according to Kruskal Wallis test followed by Steel-Dwass post hoc test ($P < 0.05$). n.s., no significantly different pairs.

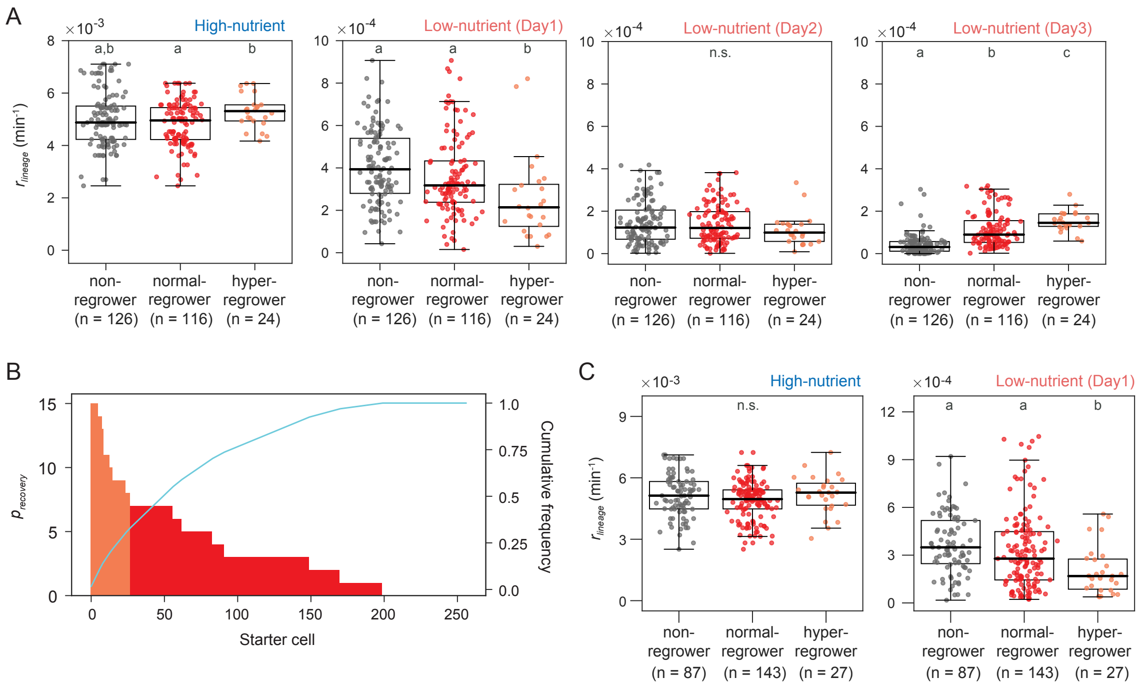

Fig. S10. Comparison of reproduction and growth history among starter cells. (A) Same as Fig. 2D and E, but the other data of the two independent experiments was used. n = 266 starter cells (in 12 microcolonies). (B) and (C) Same as Fig. 3, but the other data of the two independent experiments was used. n = 257 starter cells (in 21 microcolonies).

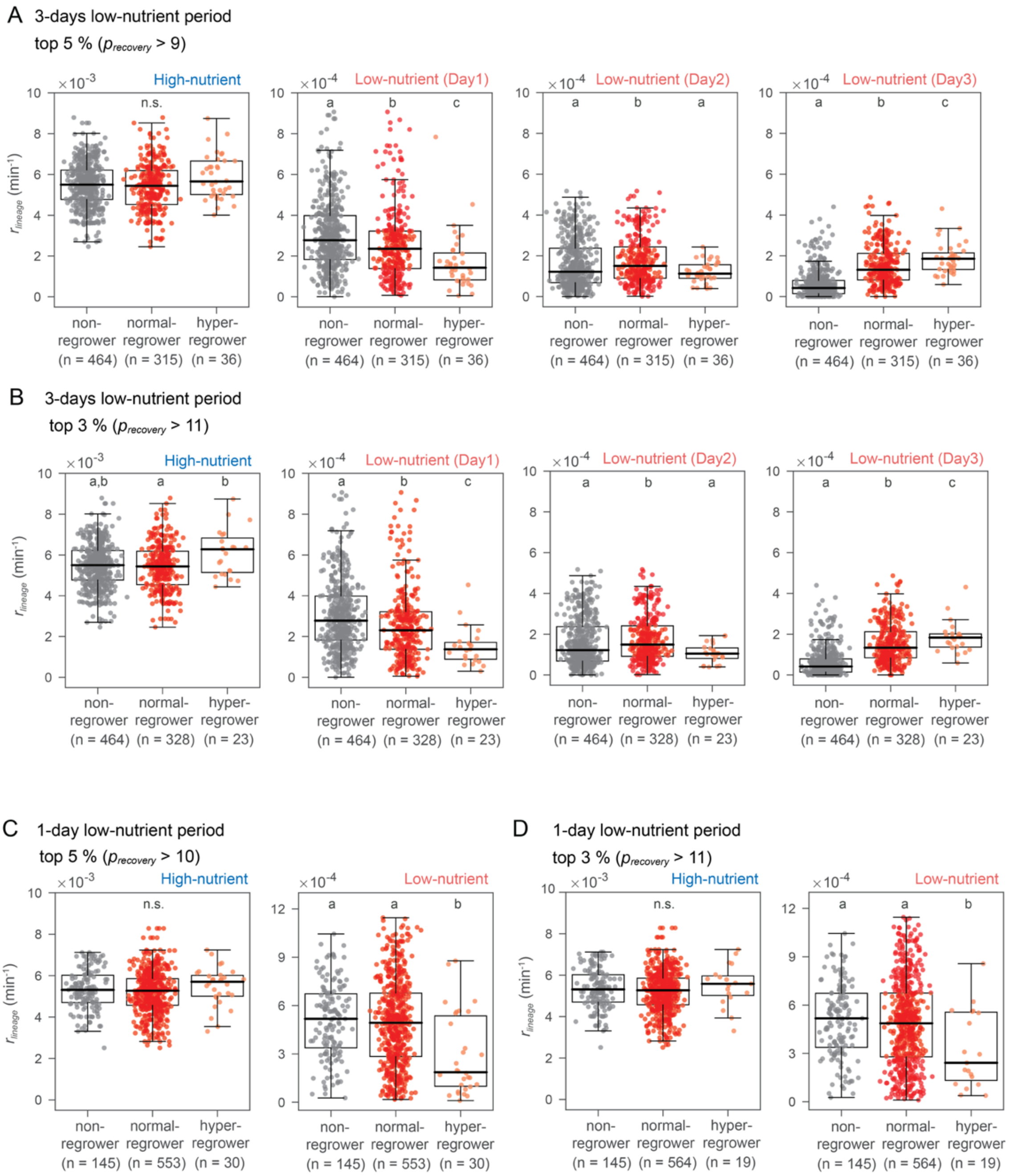

Fig. S11. Comparison of growth histories using different thresholds for hyper-regrowers. (A) and (B) Merged data of Fig. 2 and Fig. S8, but with different thresholds for hyper-regrowers. Hyper-regrowers were selected at the top 5% (A) or 3% (B) thresholds. (C) and (D) Merged data of Fig. 3 and Fig. S10, but with different thresholds for hyper-regrowers. Hyper-regrowers were selected at the top 5% (C) or 3% (D) thresholds.

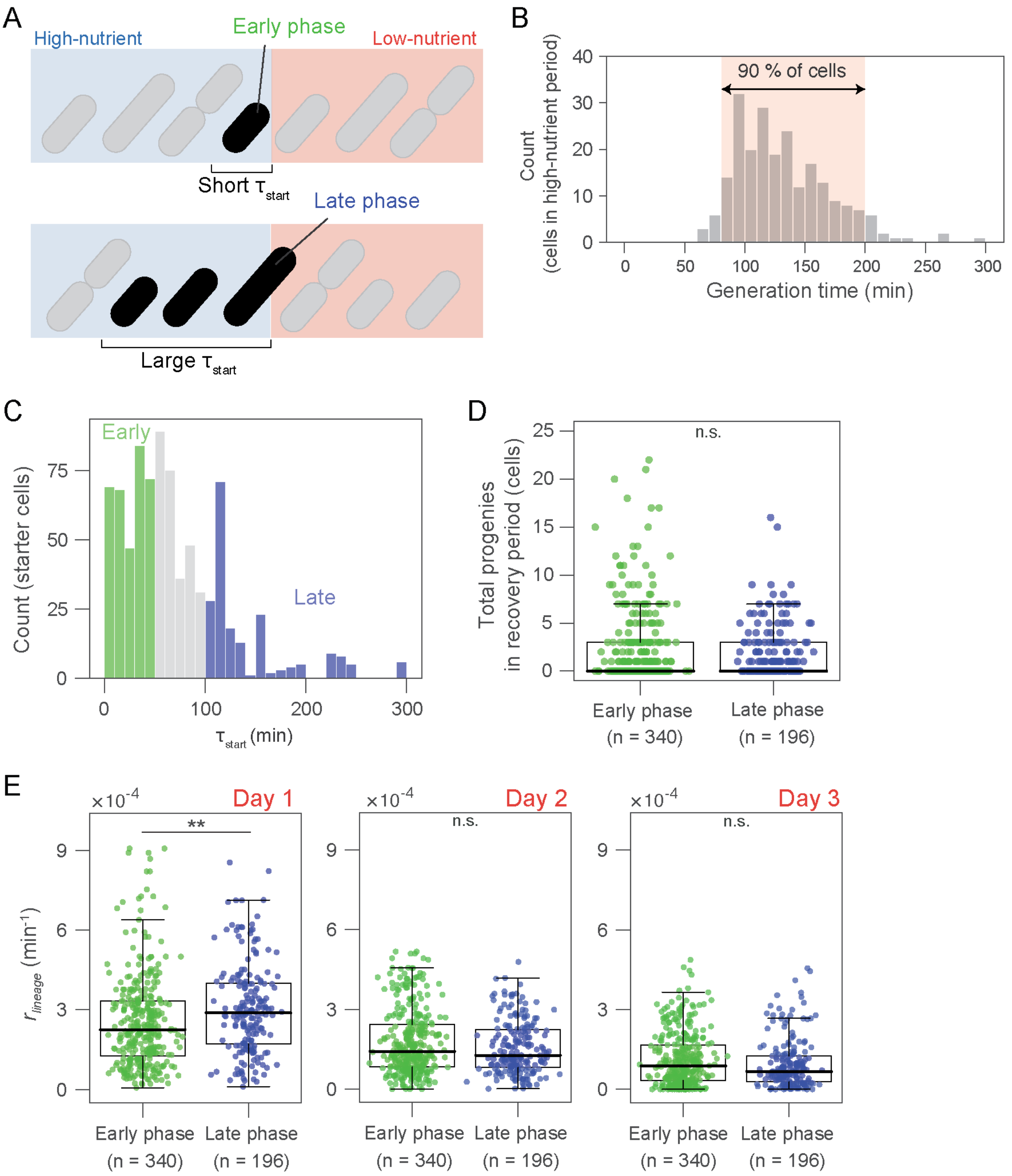

Fig. S12. Effect of the cell cycle at the transition from high- to low-nutrient periods on cellular reproduction in the recovery period. (A) A schematic illustration of the duration from the last cell division in the high-nutrient period to the beginning of the low-nutrient period (τ_{start}). We used τ_{start} for estimating cell cycle phases at the beginning of the low-nutrient period: Short τ_{start} indicates cells being in the early phase of the cell cycle, while large τ_{start} does those in the late phase. (B) Distribution of the cellular generation time (*i.e.*, the duration from birth to division) in the high-nutrient period (6 h). We analyzed 226 cells (from all 40 microcolonies in two independent experiments), 90% of

which generation time were between 80 and 200 minutes (red shaded region). (C) Distribution of τ_{start}. Data of 815 starter cells (from 40 microcolonies) after 3 days of the low-nutrient period are shown as histogram. Considering the result of (B), cells of which τ_{start} are less than 40 minutes are regarded in the early phase of the cell cycle (green), and those more than 100 minutes are regarded in the late phase (navy). (D) The number of progeny cells reproduced from two types of starter cells whose lineages were in early (n = 340) or late (n = 196) phases of the cell cycle at the beginning of the low-nutrient period. Median was shown as thick black line in each box. (E) Past $r_{lineage}$ in the low-nutrient period (day 1 to day 3) of the two types of starter cells. Median was shown as thick black line in each box. Statistical significance levels are shown according to Wilcoxon rank sum test (**, $P < 0.01$; n.s., no significant difference exists ($P > 0.05$)).

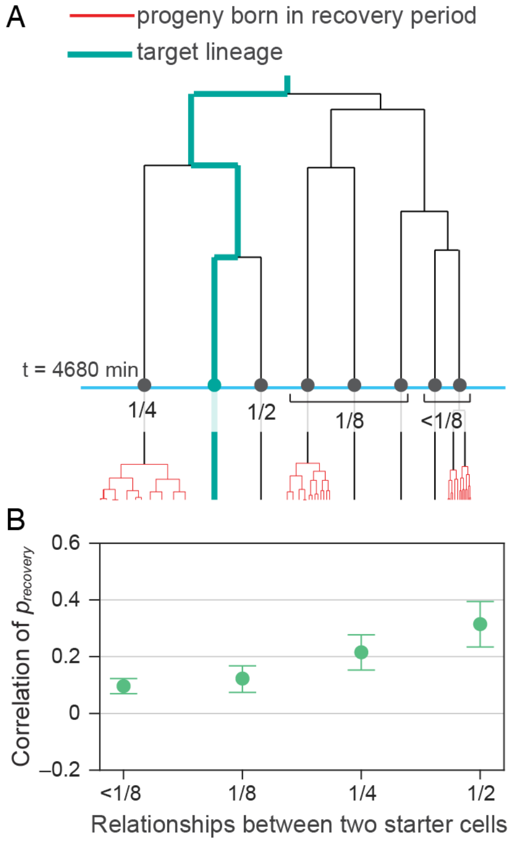

Fig. S13. Correlation between genealogical relationship and reproductivity. (A) A schematic illustration of genealogical relationships among starter cells in a lineage tree. Coefficient of relationships ($1/2^n$, where n is the number of generations to a common ancestral cell between a pair of starter cells) from a target starter cell (green circle) to the other starter cells (gray circles) are indicated. (B) Correlations of $p_{recovery}$ in pairs of starter cells. Spearman's correlation coefficients of the number of progeny cells between a pair of starter cells were calculated and plotted based on their relationships. Error bars indicate 95% bootstrap confidence interval (n = 10000).

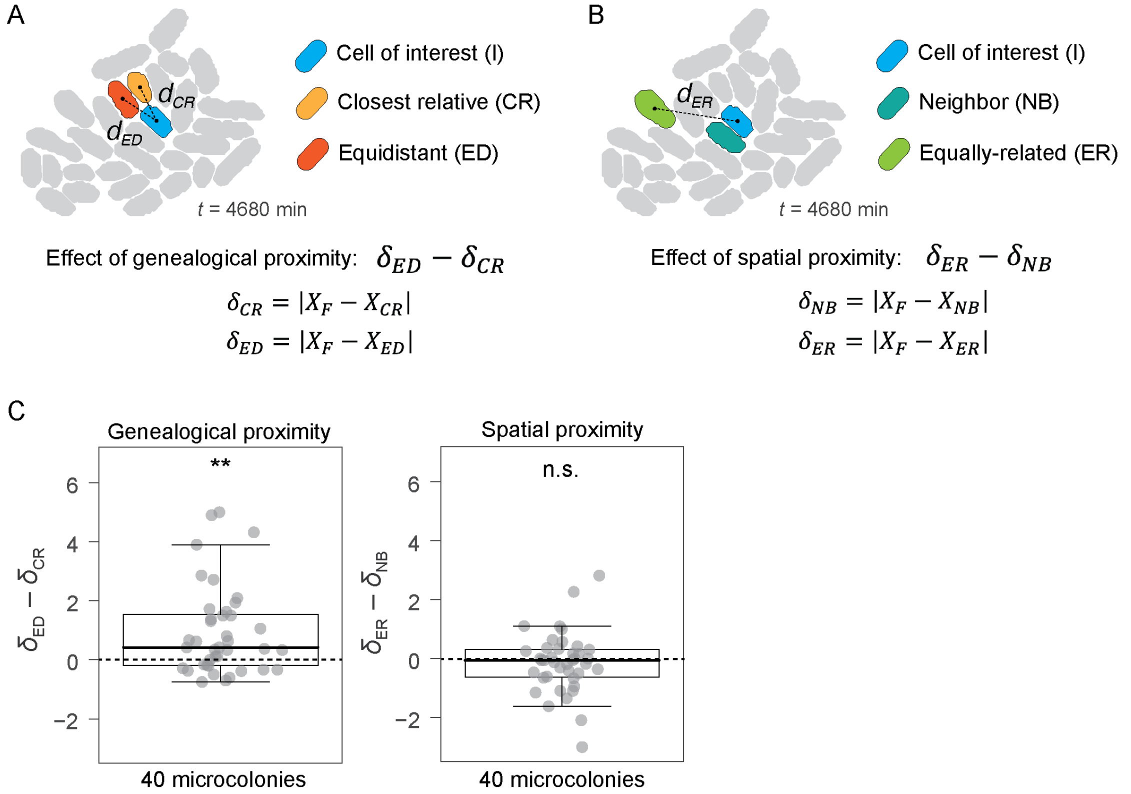

Fig. S14. Effects of genealogical proximity and spatial proximity on cellular regrowth in microcolonies. (A) A schematic illustration and equations for analyzing the dependency on genealogical proximity, taking into account the effect of spatial correlation. Dissimilarity in a given phenotype X (*i.e.*, $p_{recovery}$) between a cell of interest (blue) and its closest relative (*i.e.*, sister cell, yellow) was computed (δ_{CR}), and compared with that between the cell of interest and an "equidistant (ED)" cell (orange) (δ_{ED}), which neighbors the closest relative and has an equivalent distance from the cell of interest to that between the cell of interest and its closest relative (*i.e.*, $d_{CR} \approx d_{ED}$). If the phenotype tends to be similar between genealogically closer cells independent of their spatial proximity, $\delta_{ED} - \delta_{CR}$ should be higher than 0. (B) A schematic illustration and equations for analyzing the dependency on spatial proximity, taking into account the effect of genealogical relatedness. Dissimilarity in $p_{recovery}$ between a cell of interest (blue) and its closest neighbor (turquoise) was computed (δ_{NB}). This value was compared with that between the cell of interest and an "equally-related (ER)" cell (green) (δ_{ER}), which has the same genealogical relatedness as the closest neighbor but is located farthest from the cell of interest (*i.e.*, having the largest d_{ER}). If the phenotype tends to be similar between spatially closer cells independent of their genealogy, $\delta_{ER} - \delta_{NB}$ should be higher than 0. (C) Box plots of $\delta_{ED} - \delta_{CR}$ or $\delta_{ER} - \delta_{NB}$. The mean of each microcolony at t = 4680 min was calculated and plotted (40 microcolonies). Median values are shown as thick black lines. Statistical significance levels were estimated by one-sample Wilcoxon signed-rank test against a value of zero (**, $P < 0.01$; n.s., not significant).

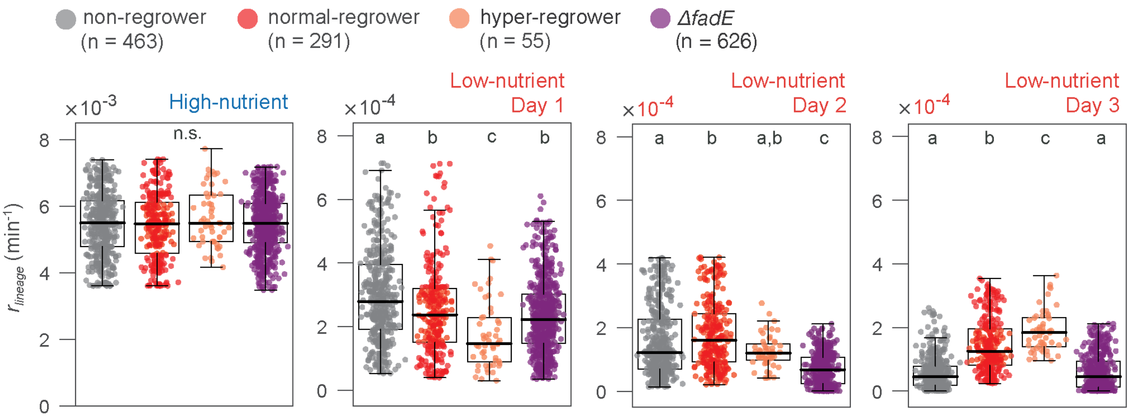

Fig. S15. Comparison of growth histories in WT and Δ*fadE* starter cells. Same as Fig. 2D and E, but the Δ*fadE* mutant data was added. Data from all 40 (WT) and 18 (Δ*fadE*) microcolonies are shown. Alphabets show statistical significance groups according to Kruskal Wallis test followed by Steel-Dwass post hoc test ($P < 0.05$).

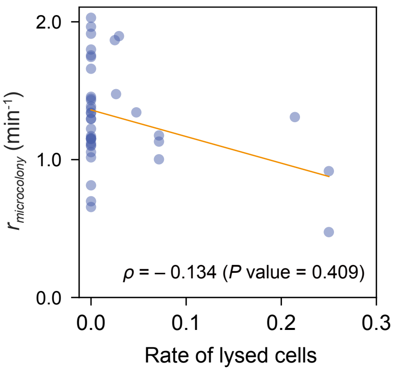

Fig. S16. Effect of lysed cells on microcolony growth in the low-nutrient period. Correlation between the rate of lysed cells (*i.e.*, total number of lysed cells per all cells present in each microcolony in the low-nutrient period) and the average size increase rate of microcolonies ($r_{microcolony}$) during the low-nutrient period. Cells that disappear in a series of time-lapse images are defined as lysed cells. $r_{microcolony}$ is calculated using the same method as for $r_{subtree}$ (See Materials and methods). Data of all 40 microcolonies with 3 days of low-nutrient period was used for the analysis. Spearman's correlation coefficient (ρ) is indicated. Note that there is no statistically significant correlation (P = 0.409).

Table S1. Oligonucleotides and templates used for PCR amplification.

Primer name	Sequence (5' to 3')	Template	Reference
**sfGFP EcoRI site fwd**	GGATCGATCCTCTAGAATTCTGATT AACTTTATAAGGAGG	pUC57 carrying a synthesized fragment of sfGFP	Addgene, USA; This study
**sfGFP rev CAT cassette complement**	ACACTTTATGCTTCCGGCTCCGCTCA TTTGTACAGTTCAT		
**CAT cassette complement fwd**	GAGCCGGAAGCATAAAGTGT	pHSG399	NBRP, Japan
**CAT cassette compleme nt rev DSR intS**	TCGATAGTTGTTAAGGTCGCACGGA AGATCACTTCGCAGA		
**DSR 500bp intS MG1655_up**	GCGACCTTAACAACTATCGAATAGC A	MG1655 chromosome	Bachmann et al., 1972[a]
**DSR 500bp intS low EcoRI site**	CGGGTACCGAGCTCGAATTCTTTGA ATAGACGAAACCGCA		
**USR 500bp intS up EcoRI site**	AGGATCGATCCTCTAGAATTTGGAG CGATTTCATCTGGCC	MG1655 chromosome	Bachmann et al., 1972[a]
**USR 440bp intS MG1655 low**	AATCATCTGGCCATTCGATGGTCAT GGAGTGCGGCTGTCA		
**PtetA_up**	CCATCGAATGGCCAGATGATTAATT	pASK-IBA3C	IBA Lifesciences, Germany
**PtetA rev sfGFP**	TTATAAAGTTAATCAGATGCCTATC ACTGATAGGGAGTGG		
**USR intS 50bp for RED/ET fwd**	TTACAGTTCGTCATGGTTCGC	pEMG carrying a P_{tetA}-*sfgfp-cat* fragment flanked by upstream and downstream regions of *intS*	This study
**DSR intS 50bp for RED/ET rev**	TTTGCACTGGATTGCAAGACT		

[a] B. J. Bachmann, Pedigrees of some mutant strains of *Escherichia coli* K-12. Bacteriological Reviews 36, 525–567 (1972).

Table S2. The number of WT cells used for calculating r_{cell} in each microcolony

microcolony_id	High-nutrient (total cells)	Low-nutrient (total cells)	Recovery (total cells)
#304	19	26	101
#203	19	44	119
#302	11	22	138
#503	3	6	4
#2402	7	16	120
#201	25	55	143
#404	15	30	127
#303	21	31	137
#501	19	30	42
#901	21	45	84
#301	35	40	375
#402	7	16	8
#2301	13	25	144
#107	13	45	66
#1602	11	30	138
#2001	11	22	122
#1501	15	52	116
#701	15	34	51
#603	15	22	105
#602	15	36	78
#902	7	26	45
#2401	3	6	22
#1701	13	47	101
#101	17	27	76
#601	7	12	10
#403	15	28	50
#801	17	31	48
#401	11	22	56
#1601	17	67	122
#102	13	21	154
#802	15	46	84
#1901	23	52	48
#504	7	8	28
#202	19	32	127
#2101	15	40	236
#702	35	84	177
#502	11	18	70
#103	19	36	169
#1401	13	49	120
#2403	19	40	323
MIN	3	6	4
MAX	35	84	375
MEAN	15.15	32.975	107.1

Movie S1 (separate file): Growth of *E. coli* WT through high-nutrient ($t \leq 6$ h), low-nutrient (6 h $\leq t \leq$ 78 h), and recovery ($t \geq 78$ h) periods. A representative microcolony is shown. The video is created by combining a series of time-lapse fluorescence images.

Movie S2 (separate file): Same as Movie S1 but through different length of low-nutrient period. A representative microcolony grows through high-nutrient ($t \leq 6$ h), low-nutrient (6 h $\leq t \leq$ 30 h), and recovery ($t \geq 30$ h) periods.

Movie S3 (separate file): Same as Movie S1 but two representative microcolonies of *E. coli fadE* mutant.
